# Human Endogenous Retroviruses in Glioblastoma Multiforme

**DOI:** 10.3390/microorganisms9040764

**Published:** 2021-04-06

**Authors:** Zihao Yuan, Yuntao Yang, Ningyan Zhang, Claudio Soto, Xiaoqian Jiang, Zhiqiang An, Wenjin Jim Zheng

**Affiliations:** 1 School of Biomedical Informatics, University of Texas Health Science Center at Houston, Houston, TX 77030, USA; zzy0012@tigermail.auburn.edu (Z.Y.); Yuntao.Yang@uth.tmc.edu (Y.Y.); Xiaoqian.Jiang@uth.tmc.edu (X.J.); 2 Texas Therapeutics Institute, Institute of Molecular Medicine, McGovern Medical School, University of Texas Health Science Center at Houston, Houston, TX 77030, USA; Ningyan.Zhang@uth.tmc.edu; 3 Department of Neurology, McGovern Medical School, University of Texas Health Science Center at Houston, Houston, TX 77030, USA; Claudio.Soto@uth.tmc.edu

**Keywords:** glioblastoma multiforme, repetitive elements, long terminal repeats, human endogenous retrovirus, brain

## Abstract

Glioblastoma multiforme (GBM) is the most aggressive and deadly brain tumor. It is primarily diagnosed in the elderly and has a 5-year survival rate of less than 6% even with the most aggressive therapies. The lack of biomarkers has made the development of immunotherapy for GBM challenging. Human endogenous retroviruses (HERVs) are a group of viruses with long terminal repeat (LTR) elements, which are believed to be relics from ancient viral infections. Recent studies have found that those repetitive elements play important roles in regulating various biological processes. The differentially expressed LTR elements from HERVs are potential biomarkers for immunotherapy to treat GBM. However, the understanding of the LTR element expression in GBM is greatly lacking. Methods: We obtained 1077.4 GB of sequencing data from public databases. These data were generated from 111 GBM tissue studies, 30 GBM cell lines studies, and 45 normal brain tissues studies. We analyzed repetitive elements that were differentially expressed in GBM and normal brain samples. Results: We found that 48 LTR elements were differentially expressed (*p*-value < 0.05) between GBM and normal brain tissues, of which 46 were HERV elements. Among these 46 elements, 34 significantly changed HERVs belong to the ERV1 superfamily. Furthermore, 43 out of the 46 differentially expressed HERV elements were upregulated. Conclusion: Our results indicate significant differential expression of many HERV LTR elements in GBM and normal brain tissues. Expression levels of these elements could be developed as biomarkers for GBM treatments.

## 1. Introduction

Glioblastoma multiforme (GBM) is the most common and most aggressive type of primary brain tumor, accounting for 47.7% of primary malignant brain tumors [1,2]. GBM is more common in males, and it appears to be sporadic without any genetic predisposition [3]. In the U.S. alone, 12,120 people in 2016, 13,010 in 2018, and 13,010 in 2019 were diagnosed with GBM. People diagnosed with GBM have a 5-year survival rate of less than 6% [1,4]. Risk factors for GBM include exposure to ionizing radiation [5], certain use of electronics [6,7,8], and infection by viruses such as human cytomegalovirus (HCMV) [9,10,11]. The biological mechanism of GBM remains a topic of controversy [12,13]. Therapy for GBM remains challenging due to the lack of efficient biomarkers and drug targets [14]. Searching for new biomarkers and drug targets to treat such a devastating disease is imperative and could have a significant impact on patient survival.

One potential source of biomarkers and drug targets is human endogenous retroviruses (HERVs) and their long terminal repeat sequences (LTRs). HERVs are believed to be relics of exogenous retroviruses integrated into the human genome throughout evolution [15]. They are major contributors to repetitive elements in the human genome. HERVs can be systematically classified into at least five groups (ERV1, ERV2, ERV3, ERV4, and endogenous lentivirus). Among them, only ERV1, ERV2, and ERV3 can be traced in the human genome [16]. HERVs and related retrotransposons account for about 8% of human genomic DNA [17,18]. HERVs are typically composed of *GAG*, *POL*, and *ENV* regions sandwiched between two LTRs [15,19,20], which are 330–1328 bp long [21,22,23]. Throughout the lengthy co-evolution, HERVs can affect host biological processes. Their effects are mediated by various genomic elements, such as alternative splicing sites, enhancers, poly-A signals, promoters, and repressors [24,25,26]. HERVs have been shown to synthesize and express unique proteins or even virus-like particles [27,28,29,30]. In some studies, HERV proteins have been shown to perform important biological functions, such as triggering or regulating host immune responses [31,32,33,34]. The transcripts from HERV-K *HML-2* have been found to be associated with a number of cancers, such as melanoma [35], leukemia and lymphoma [36], and tumors of the breast [37,38], testis [37], and ovary [38]. Expression of the HERV-E family of retrotransposable elements has been found to be correlated with prostate, kidney, ovarian, and uterine cancers [39,40]. HERV-H sequences were found to be overexpressed in colorectal carcinogenesis [41]. In addition, the results of more recent studies indicated that HERVs are associated with neurological disorders such as multiple sclerosis [42], amyotrophic lateral sclerosis [43], and schizophrenia [44]. These observations triggered the reevaluation of the importance of HERVs in disease [29,38,45] and their potential role as biomarkers and drug targets [46].

However, despite sporadic reports on the roles of some HERVs in brain tumors [47,48,49,50], a comprehensive understanding of the correlation between HERVs and glioblastoma is lacking. Although HERV LTRs can drive the expression of retroviral proteins that may be involved in various biological processes and can serve as biomarkers unique to GBM, no study has been carried out to focus on understanding the roles of LTRs, especially HERV LTRs, in GBM. The traditional study of repetitive elements via data-driven approaches remains challenging due to the ambiguity of mapping short reads to repetitive genomic sequences. The availability of the vast amount of next-generation sequencing data enabled us to characterize the landscape of the expression of HERVs and repetitive elements at an unprecedentedly comprehensive level [51], and breakthroughs in algorithms have made the understanding of the repetitive element expression landscape possible [52,53,54]. In this work, we analyzed and identified the differential expression of LTRs, including those of HERVs in GBM and normal brain tissues. The upregulated protein-coding HERVs in GBM may generate protein markers that are unique to GBM, suggesting their potential as therapeutic targets for GBM treatment.

## 2. Materials and Methods

RNA-Seq data from 111 GBM tissue studies, 30 GBM cell line studies, and 45 normal brain tissues studies were obtained from public databases such as the NCBI Sequence Read Archive (SRA: https://www.ncbi.nlm.nih.gov/sra, accessed on 5 April 2021) and the Gene Expression Omnibus (GEO: https://www.ncbi.nlm.nih.gov/geo/, accessed on 5 April 2021). Sample accessions are listed in Supplementary Table S1.

The reads were first trimmed using Trimmomatic to remove ambiguous nucleotides (N’s), extremely short reads (<30 nt), and low-quality bases with a sliding window size of 4 [55]. For quality control, the trimmed reads were mapped to the human genome (hg38) via Bowtie with the following parameters: —chunkmbs 500 −m 1 −S [56]. The resulting Sequence Alignment Map (SAM) files were converted, sorted, and indexed via SAMtools [57]. Indexed SAM files were processed by RepEnrich with default parameters [52], a software specifically designed to identify and quantify the expression of repetitive elements. Next, the EdgeR package [58] was used to analyze the expression of repetitive elements in GBM and normal brain tissues to identify significant differential expression. Significant differential expression was defined as differences with *p*-value < 0.05 and |fold change| ≥ 2 [59]. HERVs were filtered and analyzed based on the differential expression of their repetitive elements. The corresponding heatmap was plotted with Morpheus [60].

Phylogenetic analyses were conducted using differentially expressed LTR sequences. Sequence alignments were conducted using ClustalW [61] with default parameters. The phylogenetic tree was constructed using MEGA7 software [62] and the maximum likelihood method. The best model GTR + I + G was selected by Prottest 3.2.1 based on alignment results [63]. The phylogenetic tree was evaluated with 1000 bootstrap replicates.

The expressed transcripts within 1 kilobase pairs (kbp) of the differentially expressed LTRs were identified in the UCSC Genome Browser [64]. Among these, those that were differentially expressed were identified and plotted in GEPIA from TCGA [65]. The positions of the differentially expressed LTR elements on the chromosome were plotted using ChromoMap [66]. Genes interacting with differentially expressed repetitive elements via long-range chromatin interactions were identified using the 3D Interaction Viewer and Database (3DIV) [67].

A disease pathway enrichment analysis was performed on the identified neighboring genes using the WEB-based GEne SeT AnaLysis Toolkit (WebGestalt) [68] based on the gene–disease association database Disgenet [69]. The results of the analysis were plotted using the R script sp_enrichmentPlot.sh (https://github.com/Tong-Chen/s-plot/blob/master/sp_enrichmentPlot.sh, accessed on 5 April 2021). In addition, DrugBank (https://go.drugbank.com, accessed on 5 April 2021) was used to search approved drugs that target identified differentially expressed genes in proximity to the HERVs that show differential expression in GBM.

## 3. Results

**Figure 1:**
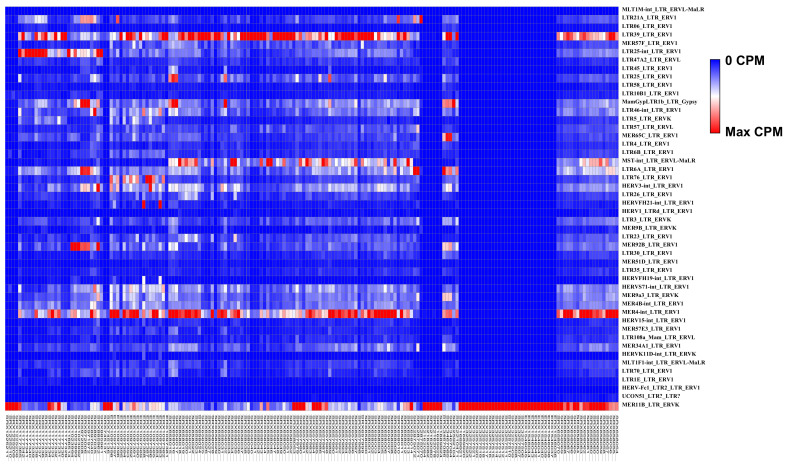
The heatmap of differentially expressed long terminal repeat (LTR) elements in glioblastoma multiforme (GBM) compared to normal brain samples. Each row represents the expression of an LTR element, while each column corresponds to an individual sample. GBM samples are highlighted in pink, and normal brain samples are in green. The color represents the expression level of LTR elements as measured by counts per million (CPM) for each sample, ranging from the highest (red) to the lowest (blue) level.

**Figure 2:**
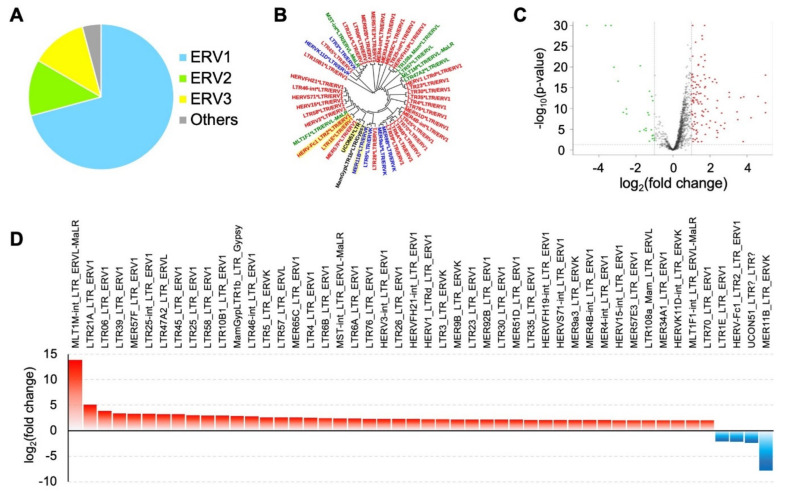
Differential expression of repetitive elements in GBM and normal brain samples. (**A**) The major categories (ERV1, ERV2, ERV3, and others) of LTR elements differentially regulated in GBM and normal brain samples. (**B**) Phylogenetic analysis of the LTR elements differentially expressed in GBM. The phylogenetic tree was constructed by the maximum likelihood method with GTR + I + G and with a bootstrap value of 1000. Red: ERV1; blue: ERVK; green: ERVL; black: others. (**C**) The volcano plot for differentially expressed repetitive elements in GBM. The x-axis shows the fold change in the expression level of repetitive elements in GBM. The y-axis represents the significance (*p*-value). The significant (*p*-value < 0.05 and |fold change| ≥ 2) up (red) and down (green) elements as well as insignificant elements (black) are plotted. (**D**) The bar chart for LTR elements differentially expressed in GBM. The y-axis represents the fold change of upregulated (red) and downregulated (blue) elements in GBM.

By sifting through RNA-Seq data that are mapped to the repetitive “junk DNA” regions of the human genome, which are ignored in a typical RNA-Seq data analysis, we identified 137 repetitive elements differentially expressed in GBM and normal brain tissues. Of these, 48 LTRs were significantly differentially expressed (|fold change| ≥ 2, *p*-value < 0.05). Furthermore, 46 of the differentially expressed LTRs can be classified as belonging to HERVs, 34 of which are members of the ERV1 superfamily (Figure 1 and Figure 2A,B).

Among the differentially expressed LTRs belonging to HERVs, 44 were upregulated and 4 were downregulated (Table 1, Figure 2C,D). Among these upregulated LTRs, 32 can be classified as ERV1s (Figure 2D), suggesting that this superfamily could be highly relevant to GBM. The most upregulated ERV1s were MLT1M-int_LTR_ERVL-MaLR, LTR21A_LTR_ERV1, and LTR06_LTR_ERV1. The next most upregulated were elements belonging to the subfamilies of ERV3 (6 of 44) and ERV2 (5 of 44). The large number of ERV1s upregulated in GBM suggests that ERV1s may serve as novel targets in future GBM therapies, especially those with an adequate amount of expression in CPM (counts per million), such as LTR21A_LTR_ERV1 (normal brain (NB) CPM, 48.57; GBM CPM, 249.19; GBM vs. NB fold changes 5.13; *p*-value < 0.05) or LTR06_LTR_ERV1 (normal brain CPM, 9.29; GBM CPM, 36.37; GBM vs NB fold changes 3.91; *p*-value < 0.05).

In contrast, a much smaller proportion of LTR elements were expressed at lower levels in GBM than in normal brain tissues. Only four LTR elements, including two ERV1s (LTR1E_LTR_ERV1 and HERV-Fc1_LTR2_LTR_ERV1), were downregulated in GBM tissues. The phylogenetic analysis showed that those downregulated ERV1s are closely related, belonging to the same phylogenetic clade (Figure 2B). This result indicates a common origin or biological function of those elements. In summary, our results show that the expression levels of LTRs, especially ERV1s, are ubiquitously higher in GBM than in normal brain tissues. These results suggest a potential functional association between the expression of HERVs and GBM. The proteins encoded by these elements could be uniquely expressed and present in GBM, suggesting a future application of those elements in drug and therapy development.

**Figure 3:**
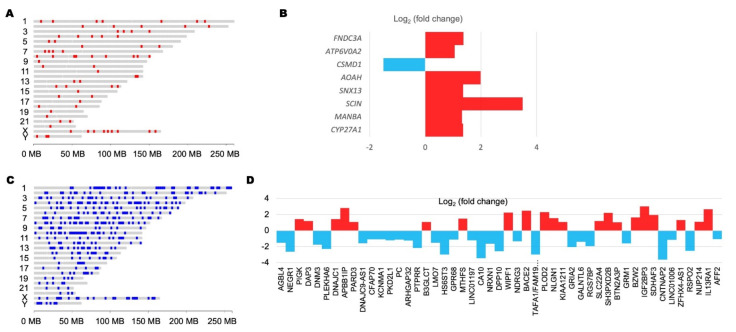
Differential expression of human endogenous retroviruses (HERVs) and the surrounding genes in GBM. (**A**) Chromosomal plot of the genomic distribution of the differentially expressed LTR21A_LTR_ERV1. (**B**) Differentially expressed genes within 1 kilobase of differentially expressed LTR21A_LTR_ERV1. (**C**) The location of the differentially expressed MER11B_LTR_ERVK plotted on chromosomes. (**D**) Differentially expressed genes within 1 kilobase of differentially expressed MER11B_LTR_ERVK.

**Figure 4:**
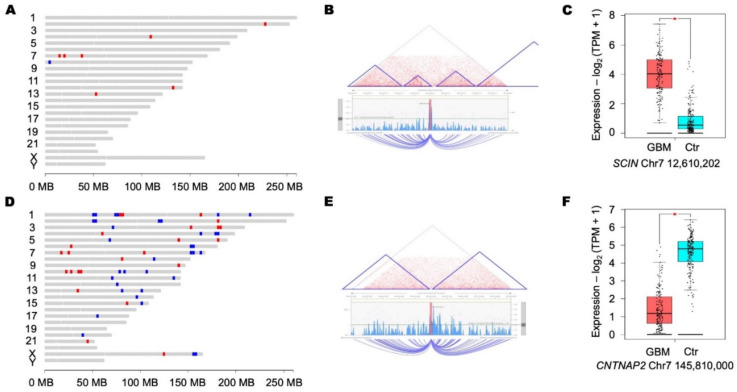
Differential expression of HERVs and neighboring genes in GBM. For the differentially expressed element LTR21A_LTR_ERV1 (chromosome 7: 12,610,202): (**A**) chromosomal plot of differentially expressed genes in spatial proximity; (**B**) potential long-range interaction predicted by 3DIV; and (**C**) the expression level of a long-range interacting gene SCIN in GBM (red, *n* = 163) and normal brain tissue (blue, *n* = 207). For differentially expressed MLT1M-int_LTR_ERVL-MaLR element (chromosome 7: 145,810,000): (**D**) chromosomal plot of differentially expressed genes in spatial proximity; (**E**) potential long-range interaction predicted by 3DIV; and (**F**) the expression level of *CNTNAP2*, identified through long-range interaction, in GBM (red, *n* = 163) and normal brain tissue (blue, *n* = 207). In (**C**,**F**), the y-axis represents the normalized gene expression level (log_2_ (TPM + 1)). * means the significant differentially expressed element between GBM and normal brain.

For the differentially expressed LTRs, there are some nearby transcripts whose expression levels also differ in GBM and normal brain tissue. The correlation could cast some light on the impact of LTR elements on the transcription profile of GBM. For LTR elements, LTR21A_LTR_ERV1 was observed to be upregulated (Figure 3A), and there were seven genes upregulated in the adjacent regions (Figure 3B). For the downregulated MER11B_LTR_ERVK elements (Figure 3C), 21 adjacent genes were upregulated and 28 were downregulated (Figure 3D). In addition, the search against the 3VD database showed that several genes or genomic elements potentially interact with those differentially expressed LTR elements through long-distance interaction and may provide some insight into gene expression regulation in GBM (Figure 4). Detailed information about the differentially expressed genes or affected genomic regions adjacent to the target LTR elements are presented in Supplementary Materials S2 and S3.

**Figure 5:**
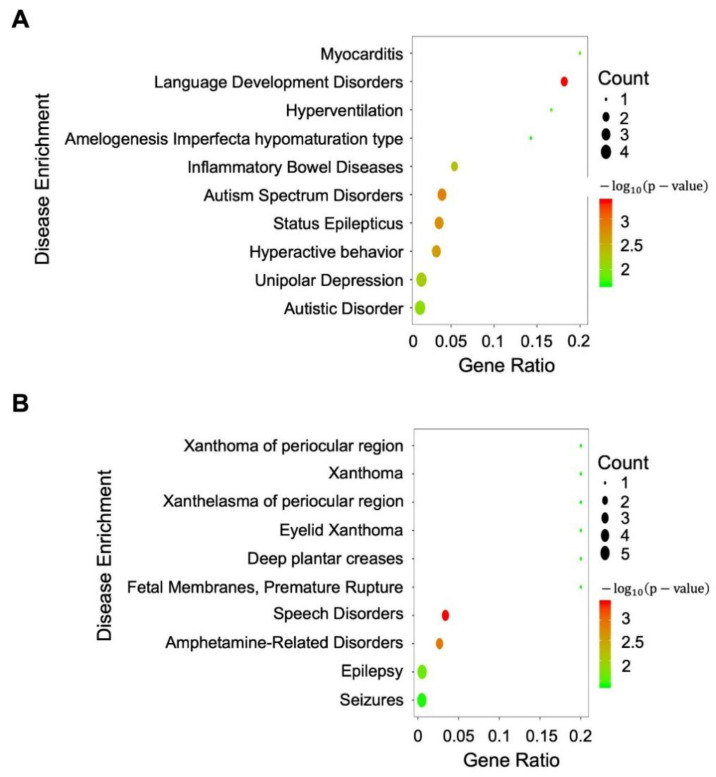
The pathway enrichment analysis of differentially expressed neighboring genes near differentially expressed HERVs. (**A**) For genes neighboring MER11B_LTR_ERVK; (**B**) for genes neighboring LTR21A_LTR_ERV1. The size of the dot represents the number of differentially expressed genes that are related to the corresponding disease identified. The color of the dot represents the confidence (*p*-value) of the enrichment.

We further analyzed the differentially expressed genes by performing a disease pathway enrichment analysis. For the genes adjacent to differentially expressed HERVs such as MER11B_LTR_ERVK or LTR21A_LTR_ERV1, a number of them are related to brain diseases such as amphetamine-related, status epilepticus, or other nerve system disorders (Figure 5). This observation raised an interesting possibility that those differentially expressed HERVs may be located in genomic regions with potentially important roles in brain dysfunctions.

We also performed a search in DrugBank to identify drugs that target these neighboring genes (Table 2). Interestingly, several of these genes identified from Hi-C data are targeted for brain functions. For example, the solute carrier family 22 member 4 (*SLC22A4*) is a transmembrane protein targeted by several drugs for brain functions. In addition, carbonic anhydrase 10 (*CA10*) encodes a carbonic anhydrase and is considered to play a role in the central nervous system, especially in brain development. Furthermore, glutamate ionotropic AMPA type subunit 2 (*GRIA2*) belongs to a family of glutamate receptors that are considered to be the predominant excitatory neurotransmitter receptors in the mammalian brain and are also targeted for brain function. All these observations point to possible future research directions to decipher whether there is any biological relevance between differentially expressed HERV elements and these neighboring genes that not only share similar gene expression patterns but are also involved in brain functions and therapies in the central nervous system.

## 4. Discussion

HERVs have been integrated into the human genome for millions of years. They can encode proteins that are differentially expressed under different disease conditions, making them potential biomarkers or therapeutic targets. Through a comparison of data from 45 non-GBM tissues, 111 GBM tissues, and 30 GBM cell culture samples, we observed that 48 LTR elements of HERVs were expressed at significantly different levels in GBM than in normal brain cells or tissues. Of these, 44 were upregulated and 4 were downregulated. Most of the upregulated HERVs in GBM (34 out of 44) belong to the ERV1 superfamily (Table 1, Figure 2D). This result is in agreement with previous research that demonstrates that ERV1 is vastly upregulated in diseases and associated cells, including psoriatic skin [70], prostate cancer cell lines [52], and osteosarcoma [71]. Thus, ERV1 could be a potential biomarker for future GBM treatments. Interestingly, the most downregulated HERV is HERV-K, which is consistent with a previous study showing that almost no HERV-Ks can be amplified from human astrocytic tumors [50]. In addition, we observed that MER4-int LTR_ERV1 and LTR39_LTR_ERV1 were expressed at different levels in both GBM and a subgroup of healthy patients. While there could be many possible reasons, further analysis would be helpful to understand this phenomenon.

We also found that the genes neighboring LTR elements were also differentially expressed in GBM and control brain samples. For example, the MLT1M-int_LTR_ERVL-MaLR elements on chromosome 7 were downregulated in GBM, along with the downregulation of the adjacent gene, *CNTNAP2* (contactin-associated protein 2). *CNTNAP2* is an important neurogenesis gene that some studies suggest is a tumor suppressor gene in glioma [72,73]. This observation could provide some clues to the mechanisms and functional implication of differentially expressed elements.

Previous research indicated that brain disorders such as disabilities in cognitive functions are frequently observed and strongly correlated in brain tumor patients [74,75]. Therefore, interfering with the expression of nervous system-related genes may indicate a potential correlation with the risk of brain tumors such as GBM. Furthermore, retroviruses can be inherited just like a regular gene, and their mutations can accumulate throughout evolution. Therefore, our observations of HERVs could cast some light on the etiology of GBM [76]. In our study, we found that a number of differentially expressed genes adjacent to differentially expressed HERVs are related to mental or central nervous system disorders. The observation indicates that differentially expressed HERVs in humans may be located in genomic regions that are important for brain functions. Furthermore, the correlated differential expression between these HERV elements and the genes involved in mental and central nervous system disorders provide a possible direction to further investigate the roles of HERVs in brain dysfunctions and even brain tumors.

Our analysis of differentially expressed genes could also provide insight into their relationship with GBM and other brain diseases such as Alzheimer’s disease (AD). Indeed, several differentially expressed proteins have been associated with AD. For example, amyloid-beta precursor protein binding family B member 1 interacting protein (APBB1) and beta secretase 2 have been involved in the modulation of APP processing and activity [77,78]. Neuroligin 1 has been shown to mediate the synaptic and memory deficits associated with AD [79]. Our study comprehensively characterized the landscape of the expression of repetitive elements, particularly HERVs, in GBM, as it differs from that of a normal brain. Our results suggest the potential application of such elements in the development of cancer therapies.

## 5. Conclusions

In our study, we comprehensively characterized the expression of repetitive elements differentially expressed in GBM and normal brain tissues. We identified 46 HERV elements, among which 43 were upregulated in GBM. The differentially expressed HERVs were also correlated with other differentially expressed genes or genomic elements nearby or through long-range genome interactions, indicating the potential functional role of HERVs in GBM. Furthermore, upregulated LTR elements of HERVs could express proteins that are unique to GBM. These could be used as future biomarkers or immunotherapy targets for GBM treatment.

## Figures and Tables

**Table 1:** LTR elements that are significantly differentially expressed (*p*-value < 0.05 and |fold change| ≥ 2) in glioblastoma multiforme (GBM) tissues, cell cultures, and normal brain (NB). The superfamily, CPM (counts per million), and fold change of the differentially expressed LTR elements in GBM and normal brain samples are presented.

LTR Elements	Superfamily	NB Mean CPM	GBM Mean CPM	Fold Changes GBM vs. NB
MLT1M-int_LTR_ERVL-MaLR	ERV3	0	0.03	13.85
LTR21A_LTR_ERV1	ERV1	48.57	249.19	5.13
LTR06_LTR_ERV1	ERV1	9.29	36.37	3.91
LTR39_LTR_ERV1	ERV1	281.42	947.75	3.37
MER57F_LTR_ERV1	ERV1	44.07	145.12	3.29
LTR25-int_LTR_ERV1	ERV1	98.72	324.11	3.28
LTR47A2_LTR_ERVL	ERV3	26.76	87.46	3.27
LTR45_LTR_ERV1	ERV1	12.9	41.61	3.23
LTR25_LTR_ERV1	ERV1	53.83	161.48	3
LTR58_LTR_ERV1	ERV1	13.2	39.2	2.97
LTR10B1_LTR_ERV1	ERV1	31.4	92.43	2.94
MamGypLTR1b_LTR_Gypsy	Gypsy	106.56	305.69	2.87
LTR46-int_LTR_ERV1	ERV1	70.41	199.2	2.83
LTR5_LTR_ERVK	ERV2	32.82	85.7	2.61
LTR57_LTR_ERVL	ERV3	49.21	127.45	2.59
MER65C_LTR_ERV1	ERV1	60.88	156.72	2.57
LTR4_LTR_ERV1	ERV1	24.56	61.49	2.5
LTR6B_LTR_ERV1	ERV1	42.14	103.63	2.46
MST-int LTR ERVL-MaLR	ERV3	132.56	320	2.41
LTR6A_LTR_ERV1	ERV1	137.45	325.28	2.37
LTR76_LTR_ERV1	ERV1	60.3	141.55	2.35
HERV3-int_LTR_ERV1	ERV1	118.4	275.59	2.33
LTR26_LTR_ERV1	ERV1	48.93	112.36	2.3
HERVFH21-int_LTR_ERV1	ERV1	22.23	50.74	2.28
HERV1_LTRd_LTR_ERV1	ERV1	6.46	14.7	2.28
LTR3_LTR_ERVK	ERV2	65.17	145.43	2.23
MER9B_LTR_ERVK	ERV2	16.12	35.17	2.18
LTR23_LTR_ERV1	ERV1	61.05	132.71	2.17
MER92B_LTR_ERV1	ERV1	96.38	209	2.17
LTR30_LTR_ERV1	ERV1	29.1	62.58	2.15
MER51D_LTR_ERV1	ERV1	11.86	25.37	2.14
LTR35_LTR_ERV1	ERV1	25.63	54.69	2.13
HERVFH19-int_LTR_ERV1	ERV1	10.32	21.85	2.12
HERVS71-int_LTR_ERV1	ERV1	122.04	256.2	2.1
MER9a3_LTR_ERVK	ERV2	100.84	210.7	2.09
MER4B-int_LTR_ERV1	ERV1	105.81	221.07	2.09
MER4-int_LTR_ERV1	ERV1	317.48	656.31	2.07
HERV15-int_LTR_ERV1	ERV1	24.28	49.96	2.06
MER57E3_LTR_ERV1	ERV1	31.62	64.73	2.05
LTR108a_Mam_LTR_ERVL	ERV3	25.92	52.95	2.04
MER34A1_LTR_ERV1	ERV1	77.33	157.29	2.03
HERVK11D-int_LTR_ERVK	ERV2	6.01	12.2	2.03
MLT1F1-int_LTR_ERVL-MaLR	ERV3	40.73	82.56	2.03
LTR70_LTR_ERV1	ERV1	37.51	75.05	2
LTR1E_LTR_ERV1	ERV1	45.06	21.22	−2.12
HERV-Fc1_LTR2_LTR_ERV1	ERV1	0.34	0.16	−2.16
MER11B_LTR_ERVK	ERV2	25,765.36	3311.14	−7.78

**Table 2:** Approved drugs targeting identified neighboring genes adjacent to differentially expressed HERV elements for brain-related functions.

Gene ID	Gene Name	DrugBank ID	Drug Name
SLC22A4	solute carrier family 22 member 4	DB00122	Choline
		DB00575	Clonidine
		DB01151	Desipramine
		DB00458	Imipramine
		DB01043	Memantine
		DB06691	Mepyramine
		DB00468	Quinine
		DB14754	Solriamfetol
CA10	carbonic anhydrase 10	DB00909	Zonisamide
GRIA2	glutamate ionotropic receptor AMPA type subunit 2	DB01351	Amobarbital
		DB00312	Pentobarbital
		DB00237	Butabarbital
		DB00241	Butalbital
		DB00306	Talbutal
		DB00418	Secobarbital
		DB00599	Thiopental
		DB00794	Primidone
		DB00849	Methylphenobarbital
		DB01174	Phenobarbital
		DB01353	Butobarbital

## Data Availability

All data and materials are publicly available as described in the Supplementary Materials S1.

## Supplementary Materials

The following are available online at https://www.mdpi.com/article/10.3390/microorganisms9040764/s1, Supplementary Materials S1: Supplementary Table S1. The list of the samples, experiments, and their accessions (SRA or GEO) used for this study. Supplementary Materials S2: The elements that are differentially regulated and related to the differentially expressed LTR21A_LTR_ERV1 in a long-range chromatin interaction. For each interaction map, the heatmap indicates the level of normalized interaction frequencies, and each triangle indicates a topological association domain. The axis indicates the interaction frequency (blue bar graph) and distance-normalized interaction frequency (magenta dots), respectively. The green line indicates the cut-off for the distance-normalized interaction frequency. The significant interactions are linked via blue arcs. Supplementary Materials S3: The elements that are differentially regulated and related to the differentially expressed MER11B_LTR_ERVK in a long-range chromatin interaction. For each interaction map, the heatmap indicates the level of normalized interaction frequencies, and each triangle indicates a topological association domain. The axis indicates the interaction frequency (blue bar graph) and distance-normalized interaction frequency (magenta dots), respectively. The green line indicates the cut-off for the distance-normalized interaction frequency. The significant interactions are linked via blue arcs.

## References

[1] Ostrom Q.T., Gittleman H., Fulop J., Liu M., Blanda R., Kromer C., Wolinsky Y., Kruchko C., Barnholtz-Sloan J.S. (2015). CBTRUS statistical report: Primary brain and central nervous system tumors diagnosed in the United States in 2008–2012. Neuro-Oncology.

[2] Ohgaki H., Kleihues P. (2005). Epidemiology and etiology of gliomas. Acta Neuropathol..

[3] Lehrer S., LaBombardi V., Green S., Pessin-Minsley M.S., Germano I.M., Rosenzweig K.E. (2011). No circulating cytomegalovirus in five patients with glioblastoma multiforme. Anticancer Res..

[4] Ostrom Q.T., Gittleman H., Truitt G., Boscia A., Kruchko C., Barnholtz-Sloan J.S. (2018). CBTRUS Statistical Report: Primary Brain and Other Central Nervous System Tumors Diagnosed in the United States in 2011–2015. Neuro-Oncology.

[5] Fisher J.L., Schwartzbaum J.A., Wrensch M., Wiemels J.L. (2007). Epidemiology of brain tumors. Neurol. Clin..

[6] Barchana M., Margaliot M., Liphshitz I. (2012). Changes in Brain Glioma Incidence and Laterality Correlates with Use of Mobile Phones–A Nationwide Population Based Study in Israel. Asian Pac. J. Cancer Prev..

[7] Deltour I., Auvinen A., Feychting M., Johansen C., Klaeboe L., Sankila R., Schüz J. (2012). Mobile Phone Use and Incidence of Glioma in the Nordic Countries 1979–2008: Consistency Check. Epidemiology.

[8] Little M.P., Azizova T.V., Bazyka D., Bouffler S.D., Cardis E., Chekin S., Chumak V.V., Cucinotta F.A., de Vathaire F., Hall P. (2012). Systematic review and meta-analysis of circulatory disease from exposure to low-level ionizing radiation and estimates of potential population mortality risks. Environ. Health Perspect..

[9] Cobbs C.S., Harkins L., Samanta M., Gillespie G.Y., Bharara S., King P.H., Nabors L.B., Cobbs C.G., Britt W.J. (2002). Human cytomegalovirus infection and expression in human malignant glioma. Cancer Res..

[10] Wrensch M., Minn Y., Chew T., Bondy M., Berger M.S. (2002). Epidemiology of primary brain tumors: Current concepts and review of the literature. Neuro-Oncology.

[11] Saddawi-Konefka R., Crawford J.R. (2010). Chronic Viral Infection and Primary Central Nervous System Malignancy. J. Neuroimmune Pharmacol..

[12] Alexander B.M., Cloughesy T.F. (2017). Adult glioblastoma. J. Clin. Oncol..

[13] Hochhalter C.B., Carr C., O’Neill B.E., Ware M.L., Strong M.J. (2017). The association between human cytomegalovirus and glioblastomas: A review. Neuroimmunol. Neuroinflammation.

[14] Lim M., Xia Y., Bettegowda C., Weller M. (2018). Current state of immunotherapy for glioblastoma. Nat. Rev. Clin. Oncol..

[15] Nelson P.N., Carnegie P., Martin J., Ejtehadi H.D., Hooley P., Roden D., Rowland-Jones S., Warren P., Astley J., Murray P.G. (2003). Demystified… Human endogenous retroviruses. Mol. Pathol..

[16] Kojima K.K. (2018). Human transposable elements in Repbase: Genomic footprints from fish to humans. Mob. DNA.

[17] Lander E.S. (2001). Initial sequencing and analysis of the human genome. International Human Genome Sequencing Consortium. Nature.

[18] Cegolon L., Salata C., Weiderpass E., Vineis P., Palù G., Mastrangelo G. (2013). Human endogenous retroviruses and cancer prevention: Evidence and prospects. BMC Cancer.

[19] Bannert N., Kurth R. (2004). Retroelements and the human genome: New perspectives on an old relation. Proc. Natl. Acad. Sci. USA.

[20] Mager D., Stoye J. (2015). Mammalian endogenous retroviruses. Microbiol. Spectr..

[21] Gilboa E., Mitra S.W., Goff S., Baltimore D. (1979). A detailed model of reverse transcription and tests of crucial aspects. Cell.

[22] Swanstrom R., DeLorbe W.J., Bishop J.M., Varmus H.E. (1981). Nucleotide sequence of cloned unintegrated avian sarcoma virus DNA: Viral DNA contains direct and inverted repeats similar to those in transposable elements. Proc. Natl. Acad. Sci. USA.

[23] Yamamoto T., De Crombrugghe B., Pastan I. (1980). Identification of a functional promoter in the long terminal repeat of Rous sarcoma virus. Cell.

[24] Jern P., Coffin J.M. (2008). Effects of Retroviruses on Host Genome Function. Annu. Rev. Genet..

[25] Leib-Mösch C., Seifarth W., Schön U., Sverdlov E. (2004). Influence of human endogenous retroviruses on cellular gene expression. Retroviruses and Primate Genome Evolution.

[26] Medstrand P., Landry J.-R., Mager D.L. (2001). Long Terminal Repeats Are Used as Alternative Promoters for the Endothelin B Receptor and Apolipoprotein C-I Genes in Humans. J. Biol. Chem..

[27] Boller K., Schönfeld K., Lischer S., Fischer N., Hoffmann A., Kurth R., Tönjes R.R. (2008). Human endogenous retrovirus HERV-K113 is capable of producing intact viral particles. J. Gen. Virol..

[28] Faff O., Murray A.B., Schmidt J., Leib-Mösch C., Erfle V., Hehlmann R. (1992). Retrovirus-like particles from the human T47D cell line are related to mouse mammary tumour virus and are of human endogenous origin. J. Gen. Virol..

[29] Andersson A.C., Svensson A.C., Rolny C., Andersson G., Larsson E. (1998). Expression of human endogenous retrovirus ERV3 (HERV-R) mRNA in normal and neoplastic tissues. Int. J. Oncol..

[30] Löwer R., Löwer J., Tondera-Koch C., Kurth R. (1993). A General Method for the Identification of Transcribed Retrovirus Sequences (R-U5 PCR) Reveals the Expression of the Human Endogenous Retrovirus Loci HERV-H and HERV-K in Teratocarcinoma Cells. Virology.

[31] Larsson E., Andersson G. (1998). Beneficial Role of Human Endogenous Retroviruses: Facts and Hypotheses. Scand. J. Immunol..

[32] Lindeskog M., Medstrand P., Blomberg J. (1993). Sequence variation of human endogenous retrovirus ERV9-related elements in an env region corresponding to an immunosuppressive peptide: Transcription in normal and neoplastic cells. J. Virol..

[33] Sauter M., Roemer K., Best B., Afting M., Schommer S., Seitz G., Hartmann M., Mueller-Lantzsch N. (1996). Specificity of antibodies directed against Env protein of human endogenous retroviruses in patients with germ cell tumors. Cancer Res..

[34] Sauter M., Schommer S., Kremmer E., Remberger K., Dölken G., Lemm I., Buck M., Best B., Neumann-Haefelin D., Mueller-Lantzsch N. (1995). Human endogenous retrovirus K10: Expression of Gag protein and detection of antibodies in patients with seminomas. J. Virol..

[35] Schiavetti F., Thonnard J., Colau D., Boon T., Coulie P.G. (2002). A human endogenous retroviral sequence encoding an antigen recognized on melanoma by cytolytic T lymphocytes. Cancer Res..

[36] Contreras-Galindo R., Kaplan M.H., Leissner P., Verjat T., Ferlenghi I., Bagnoli F., Giusti F., Dosik M.H., Hayes D.F., Gitlin S.D. (2008). Human Endogenous Retrovirus K (HML-2) Elements in the Plasma of People with Lymphoma and Breast Cancer. J. Virol..

[37] Pichon J.-P., Bonnaud B., Cleuziat P., Mallet F. (2006). Multiplex degenerate PCR coupled with an oligo sorbent array for human endogenous retrovirus expression profiling. Nucleic Acids Res..

[38] Wang-Johanning F., Liu J., Rycaj K., Huang M., Tsai K., Rosen D.G., Chen D.-T., Lu D.W., Barnhart K.F., Johanning G.L. (2006). Expression of multiple human endogenous retrovirus surface envelope proteins in ovarian cancer. Int. J. Cancer.

[39] Wang-Johanning F., Frost A.R., Jian B., Azerou R., Lu D.W., Chen D.T., Johanning G.L. (2003). Detecting the expression of human endogenous retrovirus E envelope transcripts in human prostate adenocarcinoma. Cancer.

[40] Gimenez J., Montgiraud C., Pichon J.-P., Bonnaud B., Arsac M., Ruel K., Bouton O., Mallet F. (2010). Custom human endogenous retroviruses dedicated microarray identifies self-induced HERV-W family elements reactivated in testicular cancer upon methylation control. Nucleic Acids Res..

[41] Pérot P., Mullins C.S., Naville M., Bressan C., Hühns M., Gock M., Kuhn F., Volff J.-N., Trillet-Lenoir V., Linnebacher M. (2015). Expression of young HERV-H loci in the course of colorectal carcinoma and correlation with molecular subtypes. Oncotarget.

[42] Perron H., Geny C., Laurent A., Mouriquand C., Pellat J., Perret J., Seigneurin J. (1989). Leptomeningeal cell line from multiple sclerosis with reverse transcriptase activity and viral particles. Res. Virol..

[43] McCormick A.L., Brown R.H., Cudkowicz M.E., Al-Chalabi A., Garson J.A. (2008). Quantification of reverse transcriptase in ALS and elimination of a novel retroviral candidate. Neurology.

[44] Yolken R.H., Karlsson H., Yee F., Johnston-Wilson N., Torrey E. (2000). Endogenous retroviruses and schizophrenia. Brain Res. Rev..

[45] Singh S.K. (2007). Endogenous retroviruses: Suspects in the disease world. Futur. Microbiol..

[46] Gruchot J., Kremer D., Küry P. (2019). Neural Cell Responses Upon Exposure to Human Endogenous Retroviruses. Front. Genet..

[47] Díaz-Carballo D., Klein J., Acikelli A.H., Wilk C., Saka S., Jastrow H., Wennemuth G., Dammann P., Giger-Pabst U., Khosrawipour V. (2017). Cytotoxic stress induces transfer of mitochondria-associated human endogenous retroviral RNA and proteins between cancer cells. Oncotarget.

[48] Diem O., Schäffner M., Seifarth W., Leib-Mösch C. (2012). Influence of antipsychotic drugs on human endogenous retrovirus (HERV) transcription in brain cells. PLoS ONE.

[49] Flockerzi A., Ruggieri A., Frank O., Sauter M., Maldener E., Kopper B., Wullich B., Seifarth W., Müller-Lantzsch N., Leib-Mösch C. (2008). Expression patterns of transcribed human endogenous retrovirus HERV-K (HML-2) loci in human tissues and the need for a HERV Transcriptome Project. BMC Genom..

[50] Kessler A.F., Wiesner M., Denner J., Kämmerer U., Vince G.H., Linsenmann T., Löhr M., Ernestus R.I., Hagemann C. (2014). Expression-analysis of the human endogenous retrovirus HERV-K in human astrocytic tumors. BMC Res. Notes.

[51] Zhu L., Zheng W.J. (2018). Informatics, data science, and artificial intelligence. JAMA.

[52] Criscione S.W., Zhang Y., Thompson W., Sedivy J.M., Neretti N. (2014). Transcriptional landscape of repetitive elements in normal and cancer human cells. BMC Genom..

[53] Faulkner G.J., Forrest A.R., Chalk A.M., Schroder K., Hayashizaki Y., Carninci P., Hume D.A., Grimmond S.M. (2008). A rescue strategy for multimapping short sequence tags refines surveys of transcriptional activity by CAGE. Genomics.

[54] Wang J., Huda A., Lunyak V.V., Jordan I.K. (2010). A Gibbs sampling strategy applied to the mapping of ambiguous short-sequence tags. Bioinformatics.

[55] Bolger A.M., Lohse M., Usadel B. (2014). Trimmomatic: A flexible trimmer for Illumina sequence data. Bioinformatics.

[56] Langmead B., Trapnell C., Pop M., Salzberg S.L. (2009). Ultrafast and memory-efficient alignment of short DNA sequences to the human genome. Genome Biol..

[57] Li H. (2011). A statistical framework for SNP calling, mutation discovery, association mapping and population genetical parameter estimation from sequencing data. Bioinformatics.

[58] Robinson M.D., McCarthy D.J., Smyth G.K. (2010). edgeR: A Bioconductor package for differential expression analysis of digital gene expression data. Bioinformatics.

[59] Law C.W., Alhamdoosh M., Su S., Dong X., Tian L., Smyth G.K., Ritchie M.E. (2016). RNA-Seq analysis is easy as 1-2-3 with limma, Glimma and edgeR. F1000Research.

[60] Morpheus. https://software.broadinstitute.org/morpheus.

[61] Larkin M.A., Blackshields G., Brown N.P., Chenna R., Mcgettigan P.A., McWilliam H., Valentin F., Wallace I.M., Wilm A., Lopez R. (2007). Clustal W and Clustal X version 2.0. Bioinformatics.

[62] Kumar S., Stecher G., Tamura K. (2016). MEGA7: Molecular Evolutionary Genetics Analysis Version 7.0 for Bigger Datasets. Mol. Biol. Evol..

[63] Darriba D., Taboada G.L., Doallo R., Posada D. (2011). ProtTest 3: Fast selection of best-fit models of protein evolution. Bioinformatics.

[64] Kent W.J., Sugnet C.W., Furey T.S., Roskin K.M., Pringle T.H., Zahler A.M., Haussler A.D. (2002). The Human Genome Browser at UCSC. Genome Res..

[65] Tang Z., Li C., Kang B., Gao G., Li C., Zhang Z. (2017). GEPIA: A web server for cancer and normal gene expression profiling and interactive analyses. Nucleic Acids Res..

[66] Anand L. (2019). chromoMap: An R package for Interactive Visualization and Annotation of Chromosomes. bioRxiv.

[67] Yang D., Jang I., Choi J., Kim M.-S., Lee A.J., Kim H., Eom J., Kim D., Jung I., Lee B. (2018). 3DIV: A 3D-genome Interaction Viewer and database. Nucleic Acids Res..

[68] Liao Y., Wang J., Jaehnig E.J., Shi Z., Zhang B. (2019). WebGestalt 2019: Gene set analysis toolkit with revamped UIs and APIs. Nucleic Acids Res..

[69] Piñero J., Ramírez-Anguita J.M., Saüch-Pitarch J., Ronzano F., Centeno E., Sanz F., Furlong L.I. (2019). The DisGeNET knowledge platform for disease genomics: 2019 update. Nucleic Acids Res..

[70] Lättekivi F., Kõks S., Keermann M., Reimann E., Prans E., Abram K., Silm H., Kõks G., Kingo K. (2018). Transcriptional landscape of human endogenous retroviruses (HERVs) and other repetitive elements in psoriatic skin. Sci. Rep..

[71] Ho X.D., Nguyen H.G., Trinh L.H., Reimann E., Prans E., Kõks G., Maasalu K., Le V.Q., Nguyen V.H., Le N.T.N. (2017). Analysis of the Expression of Repetitive DNA Elements in Osteosarcoma. Front. Genet..

[72] Bralten L.B.C., Gravendeel A.M., Kloosterhof N.K., Sacchetti A., Vrijenhoek T., Veltman J.A., Bent M.J.V.D., Kros J.M., Hoogenraad C.C., Smitt P.A.E.S. (2010). The CASPR2 cell adhesion molecule functions as a tumor suppressor gene in glioma. Oncogene.

[73] Sakthikumar S., Roy A., Haseeb L., Pettersson M.E., Sundström E., Marinescu V.D., Lindblad-Toh K., Forsberg-Nilsson K. (2020). Whole-genome sequencing of glioblastoma reveals enrichment of non-coding constraint mutations in known and novel genes. Genome Biol..

[74] Derks J., Reijneveld J.C., Douw L. (2014). Neural network alterations underlie cognitive deficits in brain tumor patients. Curr. Opin. Oncol..

[75] Heimans J.J., Reijneveld J.C. (2012). Factors affecting the cerebral network in brain tumor patients. J. Neuro-Oncol..

[76] Lee Y.N., Bieniasz P.D. (2007). Reconstitution of an infectious human endogenous retrovirus. PLoS Pathog..

[77] Guenette S.Y., Chen J., Jondro P.D., Tanzi R.E. (1996). Association of a novel human FE65-like protein with the cytoplasmic domain of the beta-amyloid precursor protein. Proc. Natl. Acad. Sci. USA.

[78] Solans A., Estivill X., De La Luna S. (2000). A new aspartyl protease on 21q22.3, BACE2, is highly similar to Alzheimer’s amyloid precursor protein beta-secretase. Cytogenet. Cell Genet..

[79] Camporesi E., Lashley T., Gobom J., Lantero-Rodriguez J., Hansson O., Zetterberg H., Blennow K., Becker B. (2021). Neuroligin-1 in brain and CSF of neurodegenerative disorders: Investigation for synaptic biomarkers. Acta Neuropathol. Commun..

