# Supplementary material for: Human Endogenous Retroviruses in Glioblastoma Multiforme
Source: Microorganisms. 2021 Apr 6;9(4):764. doi: 10.3390/microorganisms9040764 (PMC8067472; doi:10.3390/microorganisms9040764)
Supplement: Supplementary file 1 [file microorganisms-09-00764-s001.zip › SupplementaryMaterial/Supplementary Material 1. Supplementary Table 1.pdf]

Supplementary Table 1. The list of the samples, experiments and their accessions (SRA or GEO) used for this study.

**Accession**	**Sample Types**	**Size**
SRX658054	Non-GBM tissues	3.8Gbp
SRX658055	Non-GBM tissues	3.8Gbp
SRX658056	Non-GBM tissues	3.7Gbp
SRX658057	Non-GBM tissues	3.9Gbp
SRX658058	Non-GBM tissues	3.5Gbp
SRX658059	Non-GBM tissues	3.4Gbp
SRX658060	Non-GBM tissues	7.4Gbp
SRX658061	Non-GBM tissues	4.4Gbp
SRX658062	Non-GBM tissues	2.6Gbp
SRX658063	Non-GBM tissues	3.0Gbp
SRX658064	Non-GBM tissues	3.2Gbp
SRX658065	Non-GBM tissues	3.6Gbp
SRX658066	Non-GBM tissues	4.1Gbp
SRX658067	Non-GBM tissues	3.3Gbp
SRX658068	Non-GBM tissues	3.1Gbp
SRX658069	Non-GBM tissues	3.1Gbp
SRX658070	Non-GBM tissues	4.0Gbp
ERX1546036	Non-GBM tissues	11.3Gbp
ERX1546037	Non-GBM tissues	11.9Gbp
ERX1546038	Non-GBM tissues	12.6Gbp
ERX1546039	Non-GBM tissues	10.9Gbp
ERX1546040	Non-GBM tissues	11.7Gbp
ERX1546041	Non-GBM tissues	12.7Gbp
ERX1546042	Non-GBM tissues	12.7Gbp
ERX1546043	Non-GBM tissues	12.4Gbp

ERX1546044	Non-GBM tissues	12.7Gbp
ERX1546045	Non-GBM tissues	11.9Gbp
SRX3257506	Non-GBM tissues	2.9Gbp
SRX3257507	Non-GBM tissues	1.9Gbp
SRX3257508	Non-GBM tissues	2.7Gbp
SRX3257509	Non-GBM tissues	2.8Gbp
SRX3257510	Non-GBM tissues	2.9Gbp
SRX3257511	Non-GBM tissues	2.9Gbp
SRX3257512	Non-GBM tissues	2.3Gbp
SRX3257513	Non-GBM tissues	2.7Gbp
SRX3257514	Non-GBM tissues	2.8Gbp
SRX3257515	Non-GBM tissues	2.8Gbp
SRX3257516	Non-GBM tissues	2.7Gbp
SRX3257517	Non-GBM tissues	3.1Gbp
SRX3257518	Non-GBM tissues	2.9Gbp
SRX3257519	Non-GBM tissues	3.0Gbp
SRX3257520	Non-GBM tissues	3.0Gbp
SRX3257521	Non-GBM tissues	2.9Gbp
SRX3257522	Non-GBM tissues	2.7Gbp
SRX3257523	Non-GBM tissues	3.3Gbp
SRX1689920	GBM cell line	1.7Gbp
SRX1689921	GBM cell line	1.6Gbp
SRX2210471	GBM cell line	10.6Gbp
SRX2592104	GBM cell line	13.2Gbp
SRX3467084	GBM cell line	16.4Gbp
SRX3467085	GBM cell line	13.8Gbp
SRX3467086	GBM cell line	19.2Gbp
SRX1689924	GBM cell line	1.8Gbp
SRX1689925	GBM cell line	1.8Gbp

SRX2162078	GBM tissue	9.1Gbp
SRX2162080	GBM tissue	8.0Gbp
SRX2162082	GBM tissue	8.2Gbp
SRX2162084	GBM tissue	8.4Gbp
SRX2875070	GBM tissue	8.3Gbp
SRX2875071	GBM tissue	7.7Gbp
SRX2875072	GBM tissue	8.1Gbp
SRX658014	GBM tissue	3.3Gbp
SRX658013	GBM tissue	3.4Gbp
SRX658012	GBM tissue	2.8Gbp
SRX658011	GBM tissue	2.2Gbp
SRX658010	GBM tissue	1.8Gbp
SRX658009	GBM tissue	2.5Gbp
SRX658008	GBM tissue	2.2Gbp
SRX658007	GBM tissue	2.3Gbp
SRX658006	GBM tissue	3.0Gbp
SRX658005	GBM tissue	2.5Gbp
SRX658004	GBM tissue	2.0Gbp
SRX658003	GBM tissue	1.7Gbp
SRX658002	GBM tissue	2.1Gbp
SRX658001	GBM tissue	2.4Gbp
SRX658000	GBM tissue	2.4Gbp
SRX657999	GBM tissue	2.2Gbp
SRX657998	GBM tissue	2.7Gbp
SRX657997	GBM tissue	2.4Gbp
SRX657996	GBM tissue	2.1Gbp
SRX657995	GBM tissue	2.5Gbp
SRX657994	GBM tissue	2.2Gbp
SRX657993	GBM tissue	2.0Gbp

SRX657992	GBM tissue	2.1Gbp
SRX657991	GBM tissue	2.2Gbp
SRX657990	GBM tissue	2.5Gbp
SRX657989	GBM tissue	2.1Gbp
SRX657988	GBM tissue	3.8Gbp
SRX657987	GBM tissue	2.3Gbp
SRX657986	GBM tissue	3.0Gbp
SRX657985	GBM tissue	3.1Gbp
SRX657984	GBM tissue	3.1Gbp
SRX657983	GBM tissue	3.2Gbp
SRX657982	GBM tissue	3.5Gbp
SRX657981	GBM tissue	2.9Gbp
SRX657980	GBM tissue	2.6Gbp
SRX657979	GBM tissue	3.8Gbp
SRX658053	GBM tissue	3.5Gbp
SRX658052	GBM tissue	3.7Gbp
SRX658051	GBM tissue	3.2Gbp
SRX658050	GBM tissue	3.8Gbp
SRX658049	GBM tissue	4.3Gbp
SRX658048	GBM tissue	3.2Gbp
SRX658047	GBM tissue	3.2Gbp
SRX658046	GBM tissue	3.1Gbp
SRX658045	GBM tissue	2.7Gbp
SRX658044	GBM tissue	3.7Gbp
SRX658043	GBM tissue	2.8Gbp
SRX658042	GBM tissue	2.8Gbp
SRX658041	GBM tissue	2.9Gbp
SRX658040	GBM tissue	3.3Gbp
SRX658039	GBM tissue	3.8Gbp

SRX658038	GBM tissue	3.2Gbp
SRX658037	GBM tissue	1.8Gbp
SRX658036	GBM tissue	2.2Gbp
SRX658035	GBM tissue	2.3Gbp
SRX658034	GBM tissue	3.6Gbp
SRX658033	GBM tissue	1.7Gbp
SRX658032	GBM tissue	3.0Gbp
SRX658031	GBM tissue	2.0Gbp
SRX658030	GBM tissue	1.9Gbp
SRX658029	GBM tissue	2.4Gbp
SRX658028	GBM tissue	2.0Gbp
SRX658027	GBM tissue	1.9Gbp
SRX658026	GBM tissue	2.4Gbp
SRX658025	GBM tissue	2.7Gbp
SRX658024	GBM tissue	2.3Gbp
SRX658023	GBM tissue	2.3Gbp
SRX658022	GBM tissue	2.6Gbp
SRX658021	GBM tissue	2.4Gbp
SRX658020	GBM tissue	2.1Gbp
SRX658019	GBM tissue	2.4Gbp
SRX658018	GBM tissue	2.1Gbp
SRX658017	GBM tissue	2.6Gbp
SRX658016	GBM tissue	2.7Gbp
SRX658015	GBM tissue	2.7Gbp
ERX1097381	GBM tissue	12.5Gbp
ERX1097382	GBM tissue	22.0Gbp
ERX1097383	GBM tissue	16.7Gbp
ERX1097384	GBM tissue	32.0Gbp
ERX1097385	GBM tissue	10.2Gbp

ERX1097386	GBM tissue	13.3Gbp
ERX1097387	GBM tissue	23.5Gbp
ERX1097388	GBM tissue	6.2Gbp
ERX1097389	GBM tissue	21.1Gbp
ERX1097390	GBM tissue	21.5Gbp
ERX1097391	GBM tissue	24.6Gbp
ERX1097392	GBM tissue	9.2Gbp
ERX1097393	GBM tissue	11.4Gbp
ERX1097394	GBM tissue	17.9Gbp
ERX1097395	GBM tissue	10.7Gbp
ERX1097396	GBM tissue	18.1Gbp
ERX1097397	GBM tissue	20.1Gbp
ERX1097398	GBM tissue	16.8Gbp
SRX745136	GBM tissue	19.5Gbp
SRX745137	GBM tissue	17.9Gbp
SRX885961	GBM cell line	6.2Gbp
SRX885962	GBM cell line	6.3Gbp
SRX897035	GBM tissue	5.9Gbp
SRX897040	GBM tissue	5.8Gbp
SRX971972	GBM cell line	12.8Gbp
SRX972006	GBM cell line	15.5Gbp
SRX972008	GBM cell line	14.0Gbp
SRX1023992	GBM cell line	5.3Gbp
SRX1023993	GBM cell line	4.6Gbp
SRX1023994	GBM cell line	4.5Gbp
SRX1166514	GBM cell line	5.5Gbp
SRX1177329	GBM tissue	4.9Gbp
SRX1177330	GBM tissue	5.7Gbp
SRX1177331	GBM tissue	3.6Gbp

SRX1177332	GBM tissue	4.3Gbp
SRX1177333	GBM tissue	4.1Gbp
SRX1177334	GBM tissue	4.6Gbp
SRX1177335	GBM cell line	2.2Gbp
SRX1177336	GBM cell line	1.8Gbp
SRX1177337	GBM cell line	1.3Gbp
SRX1177338	GBM cell line	2.1Gbp
SRX1177339	GBM cell line	4.7Gbp
SRX1177340	GBM cell line	5.4Gbp
SRX1177341	GBM cell line	5.3Gbp
SRX1177342	GBM cell line	4.6Gbp
SRX1177365	GBM tissue	2.9Gbp
SRX1225208	GBM cell line	4.9Gbp
SRX1225209	GBM cell line	3.1Gbp
SRX1225210	GBM cell line	3.7Gbp
SRX1225211	GBM cell line	3.5Gbp
