# Supplementary material for: Human Endogenous Retroviruses in Glioblastoma Multiforme
Source: Microorganisms. 2021 Apr 6;9(4):764. doi: 10.3390/microorganisms9040764 (PMC8067472; doi:10.3390/microorganisms9040764)
Supplement: Supplementary file 1 [file microorganisms-09-00764-s001.zip › SupplementaryMaterial/Supplementary Materials 2. LTR21A-LTR-ERV1 and differential expression.pptx]

## Slide 1
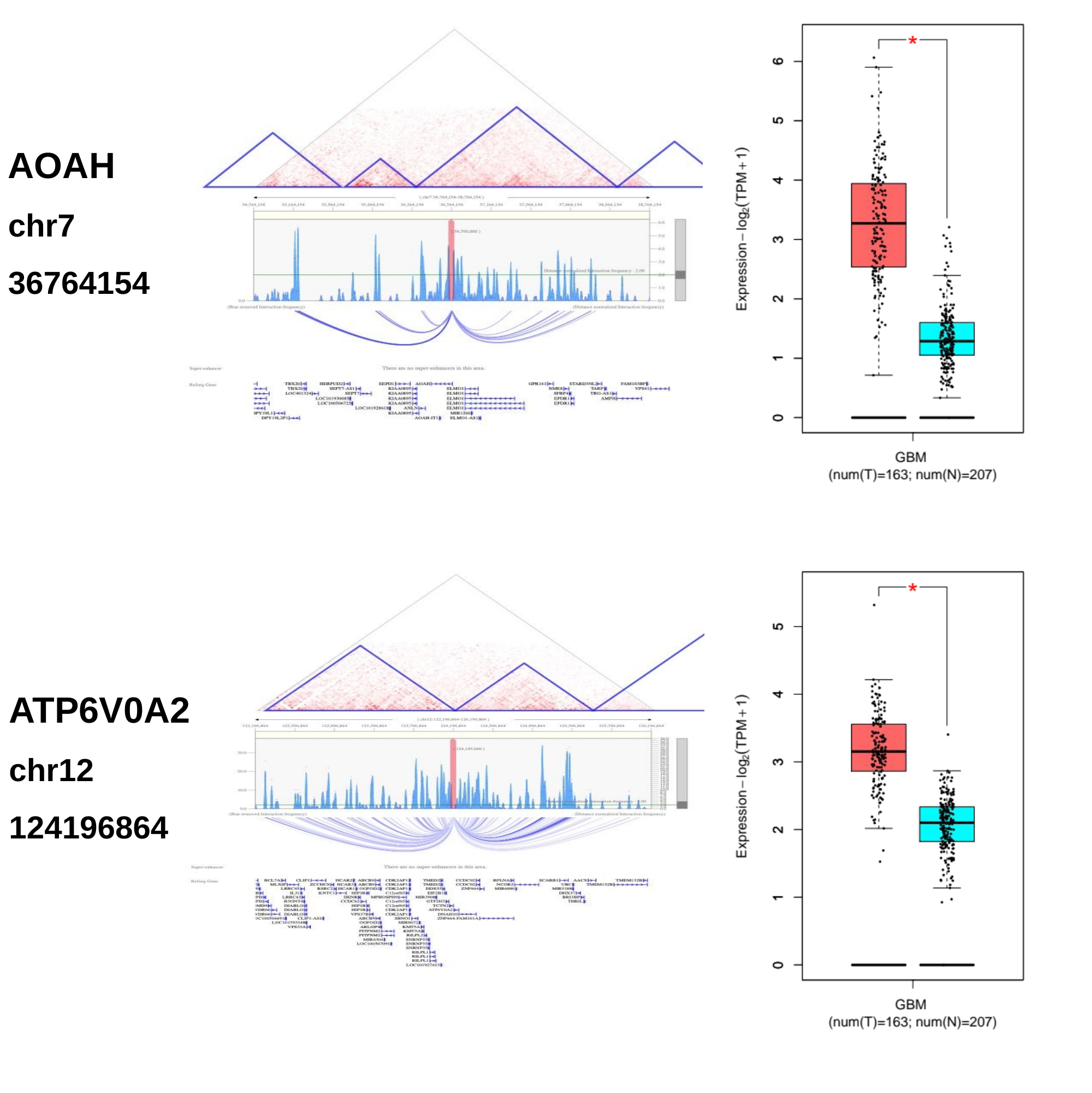

AOAH
chr7
36764154
ATP6V0A2
chr12
124196864

## Slide 2
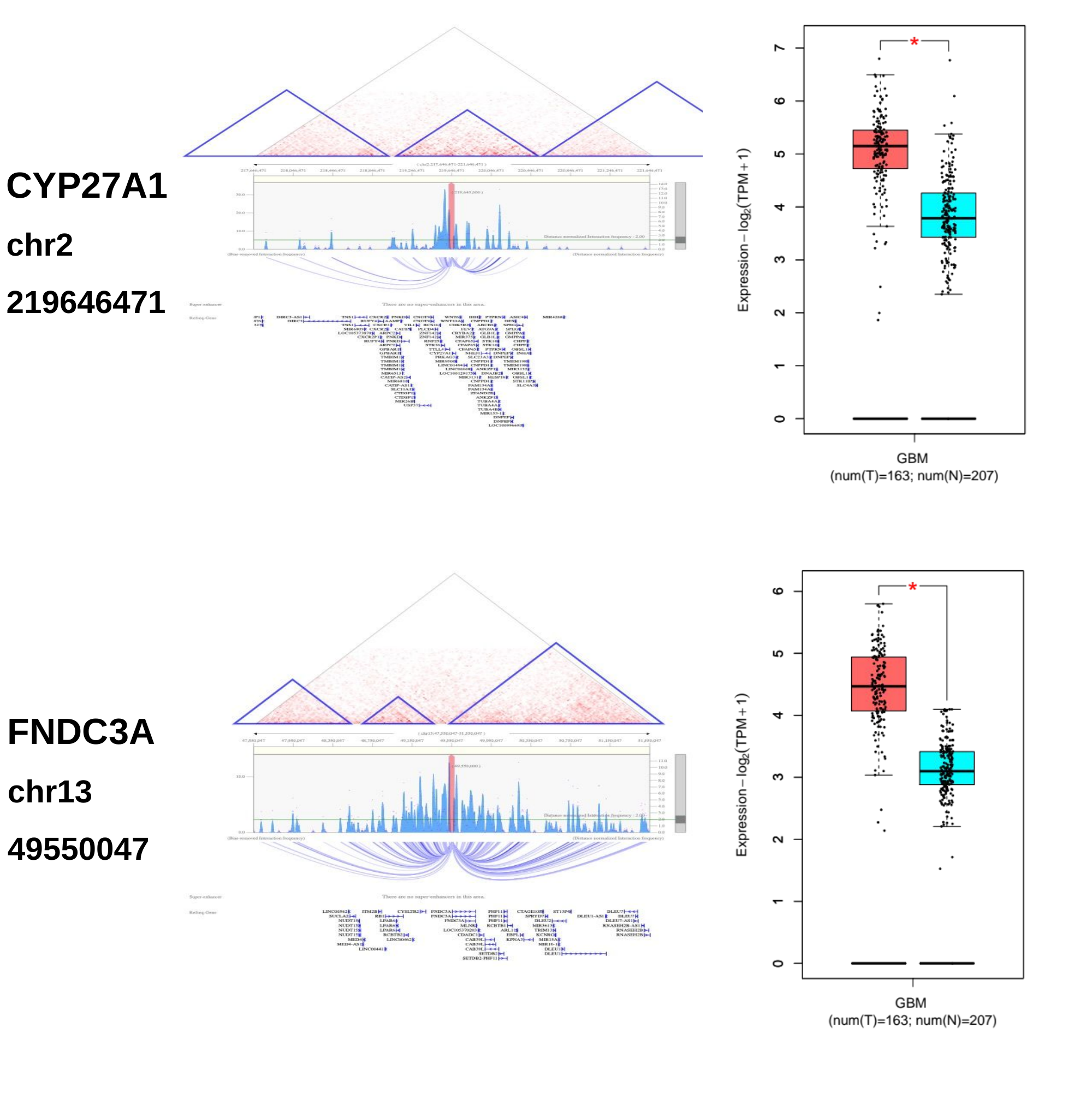

CYP27A1
chr2
219646471
FNDC3A
chr13
49550047

## Slide 3
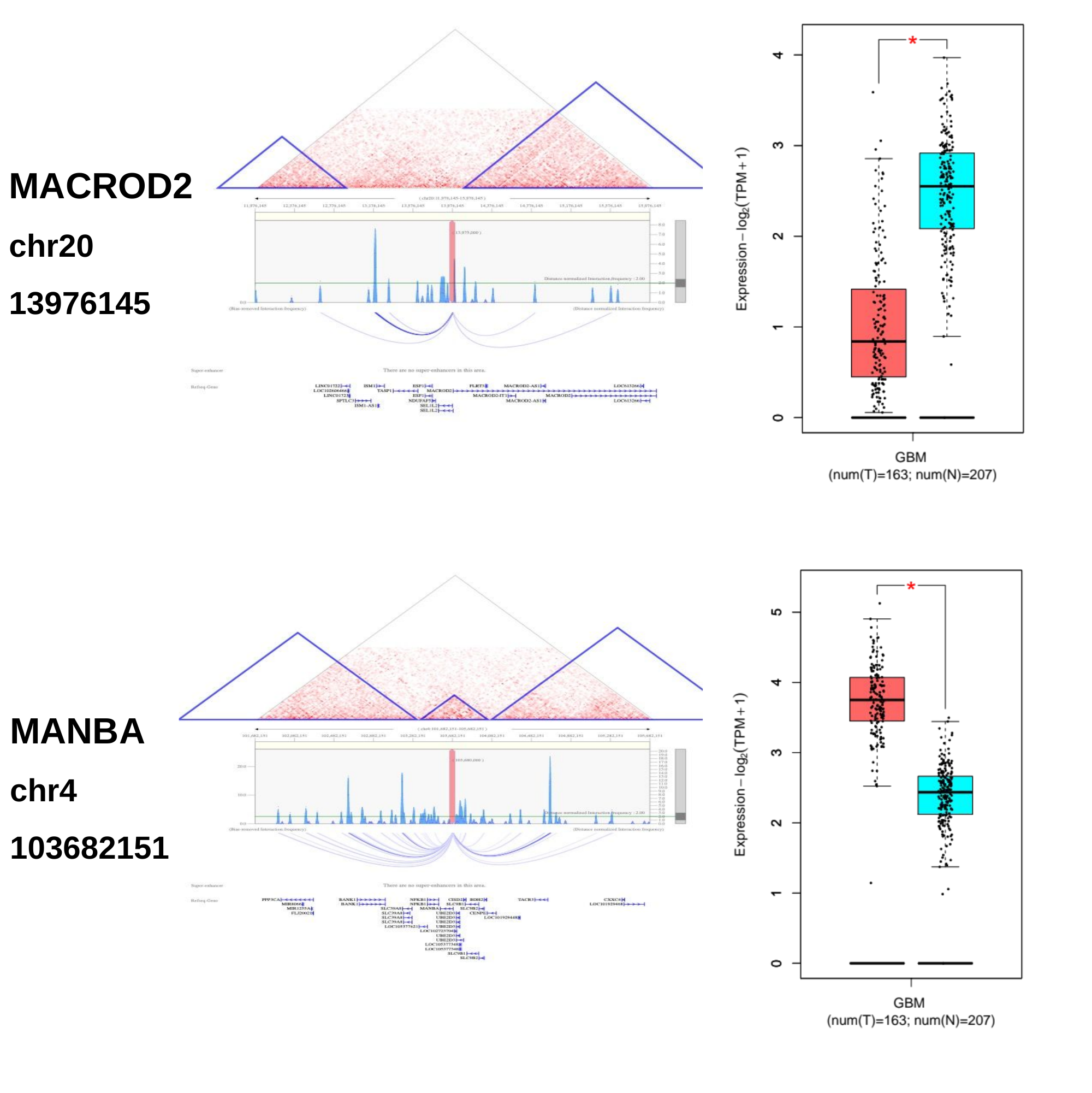

MACROD2
chr20
13976145
MANBA
chr4
103682151

## Slide 4
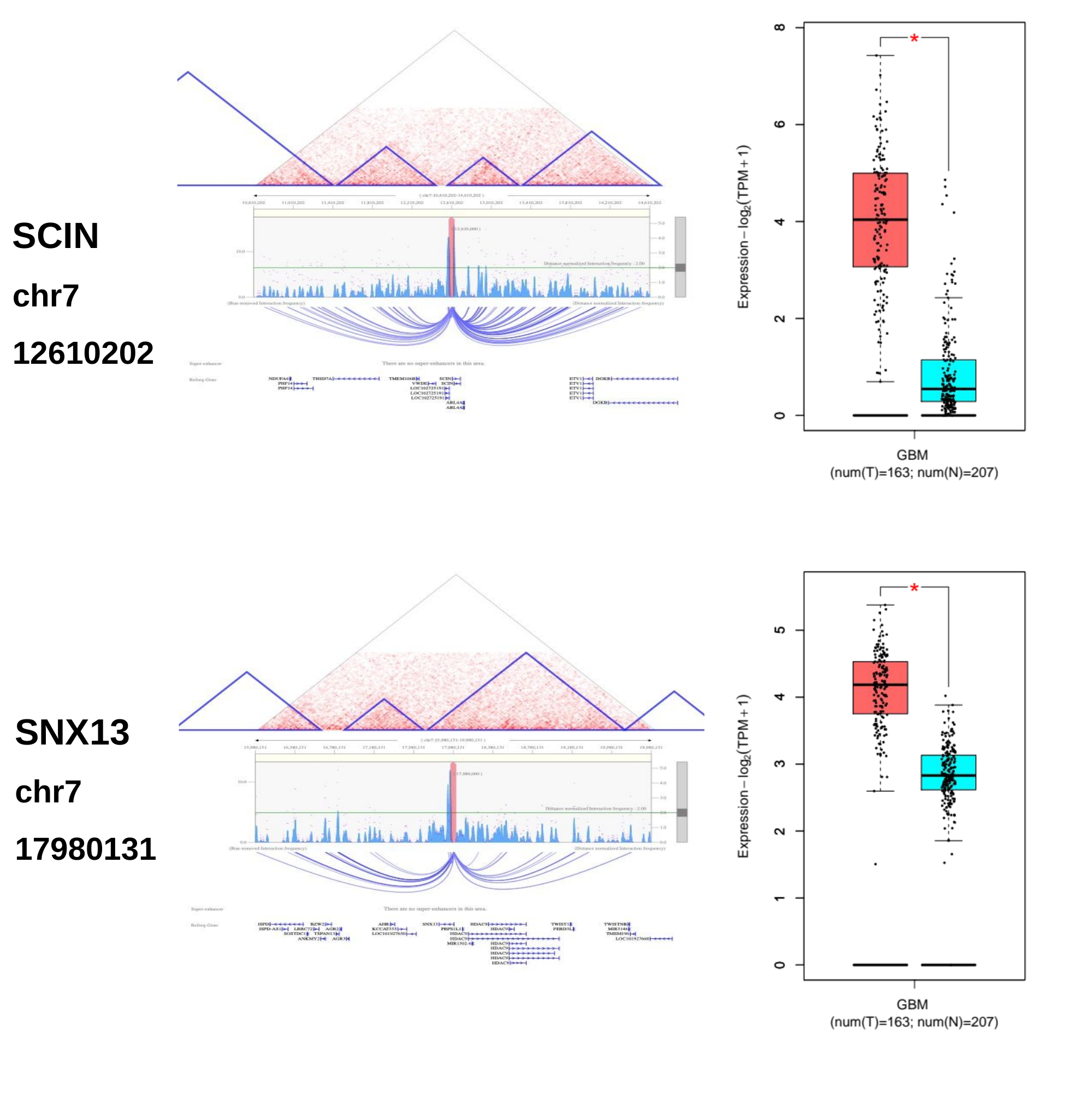

SCIN
chr7
12610202
SNX13
chr7
17980131

## Slide 5
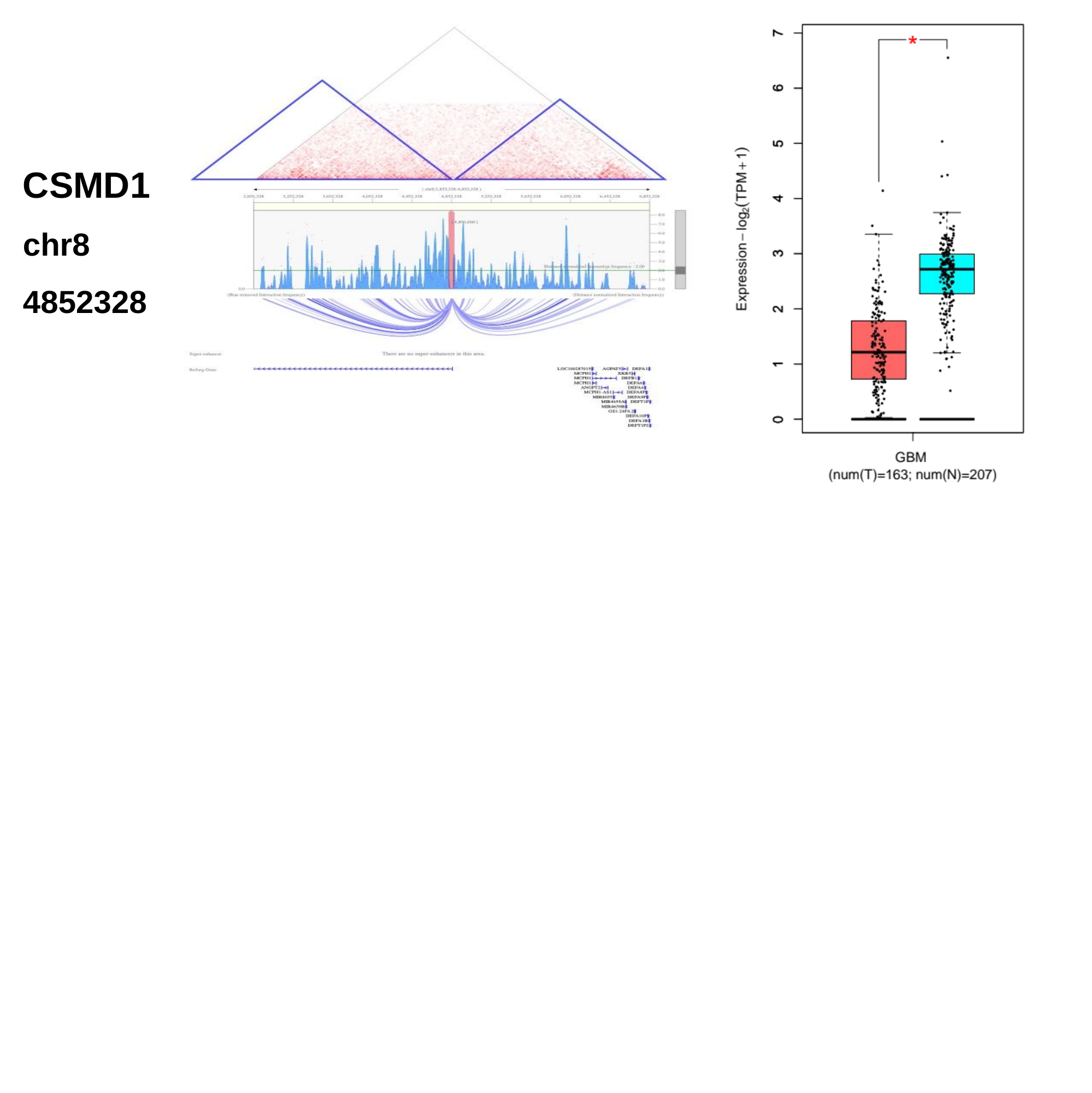

CSMD1
chr8
4852328
