# Supplementary material for: Human Endogenous Retroviruses in Glioblastoma Multiforme
Source: Microorganisms. 2021 Apr 6;9(4):764. doi: 10.3390/microorganisms9040764 (PMC8067472; doi:10.3390/microorganisms9040764)
Supplement: Supplementary file 1 [file microorganisms-09-00764-s001.zip › SupplementaryMaterial/Supplementary Materials 3. MER11B~LTR~ERVK and differential expression.pptx]

## Slide 1
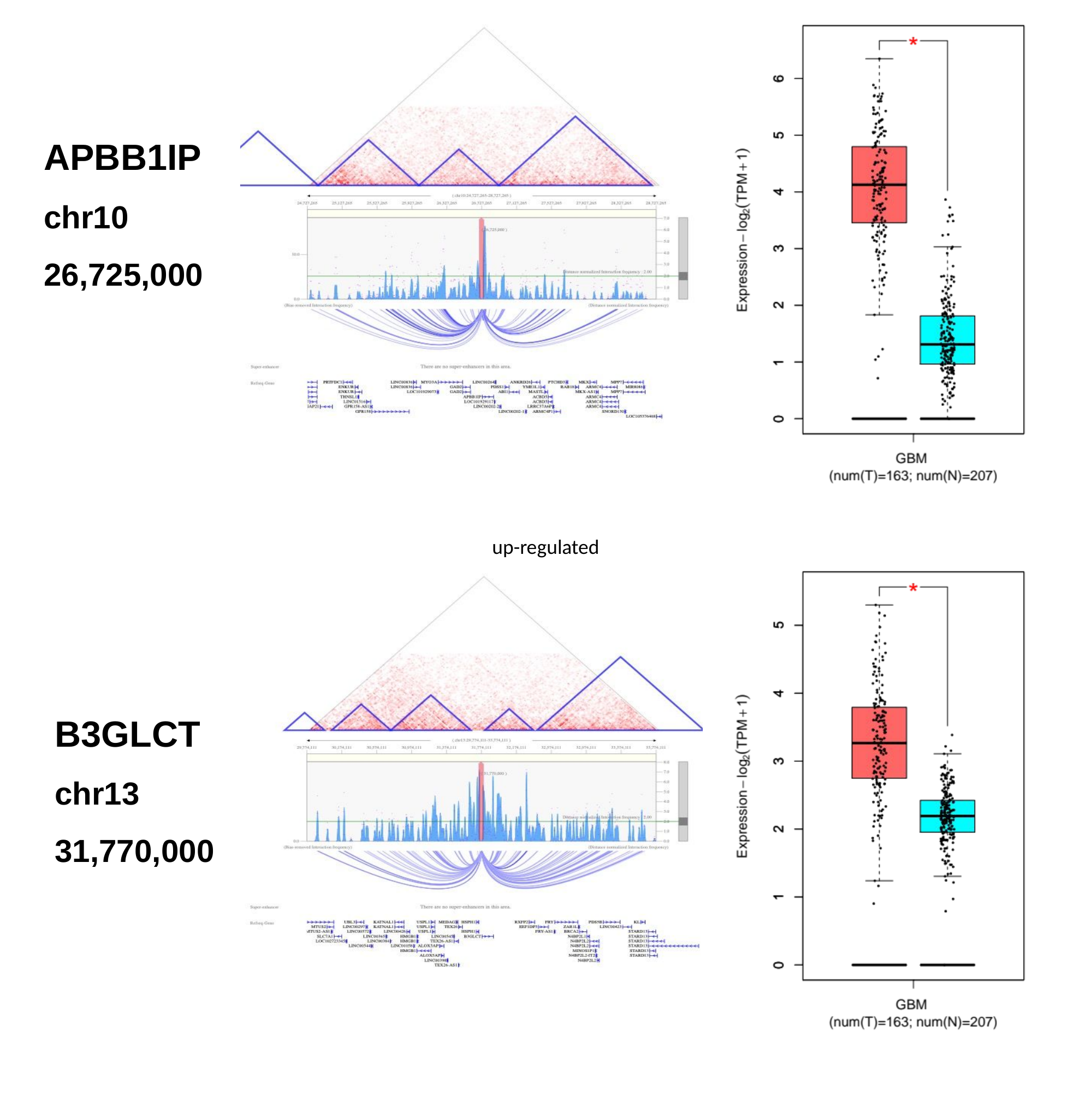

APBB1IP
chr10
26,725,000
up-regulated
B3GLCT
chr13
31,770,000

## Slide 2
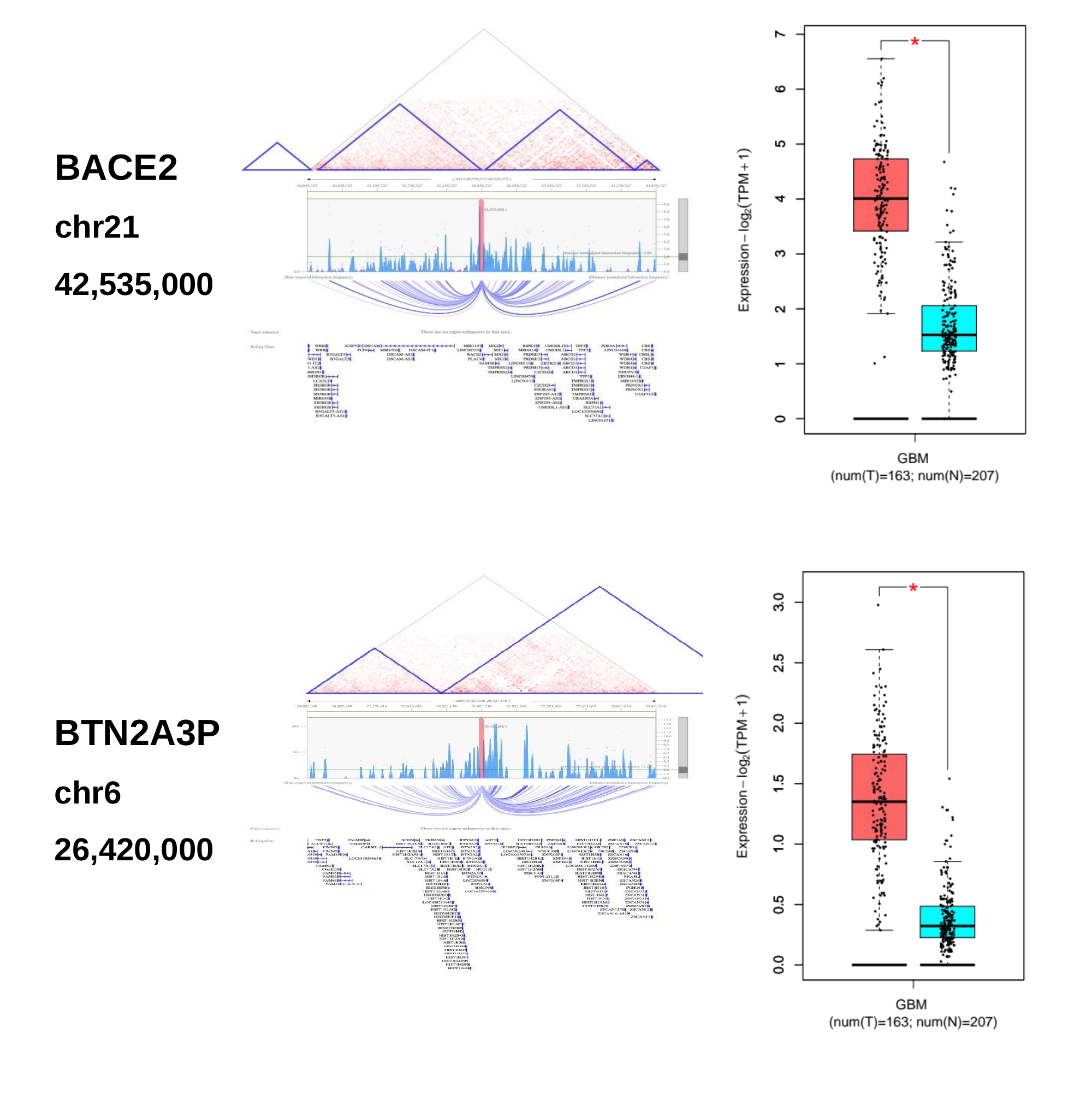

BACE2
chr21
42,535,000
BTN2A3P
chr6
26,420,000

## Slide 3
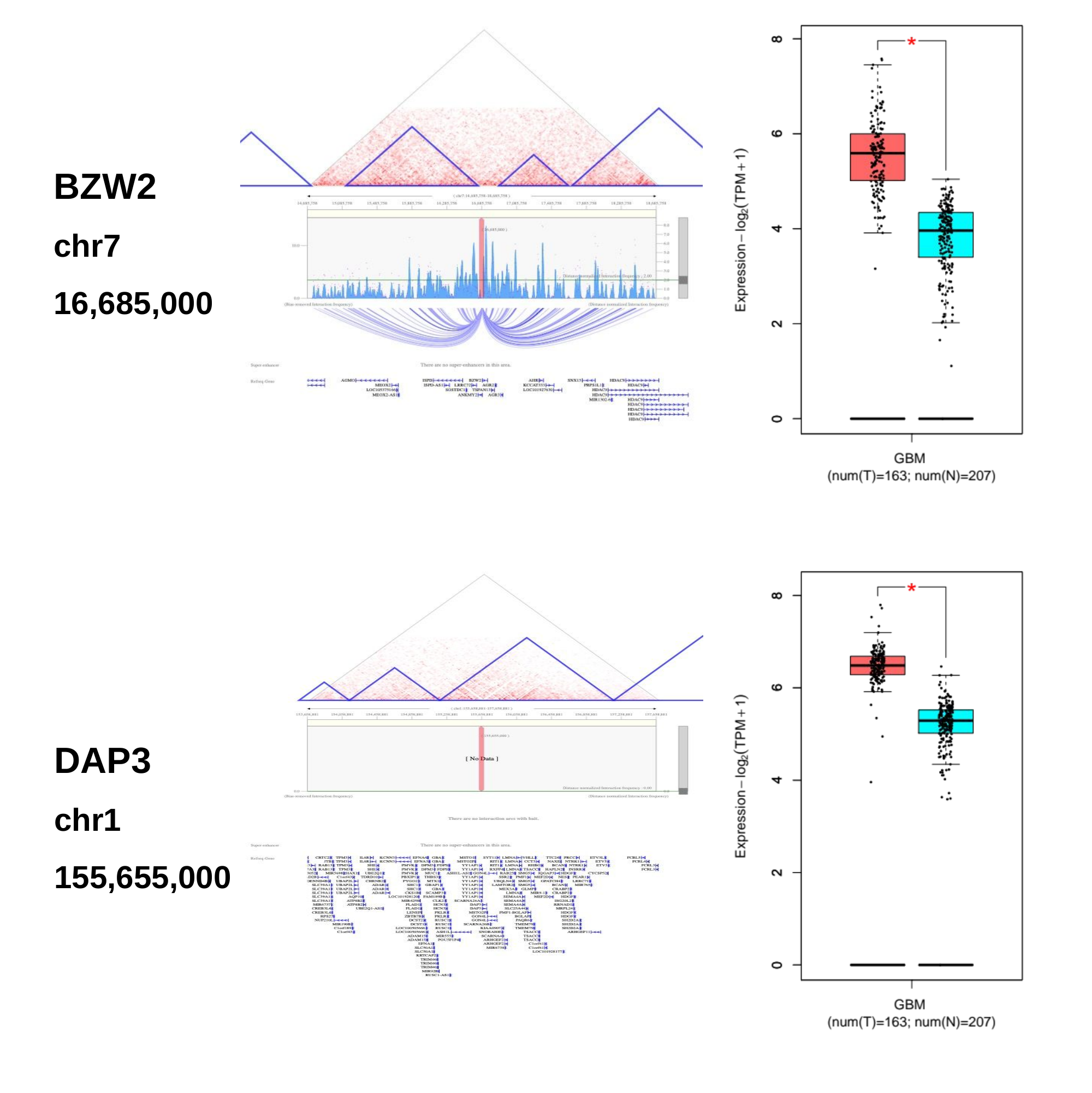

BZW2
chr7
16,685,000
DAP3
chr1
155,655,000

## Slide 4
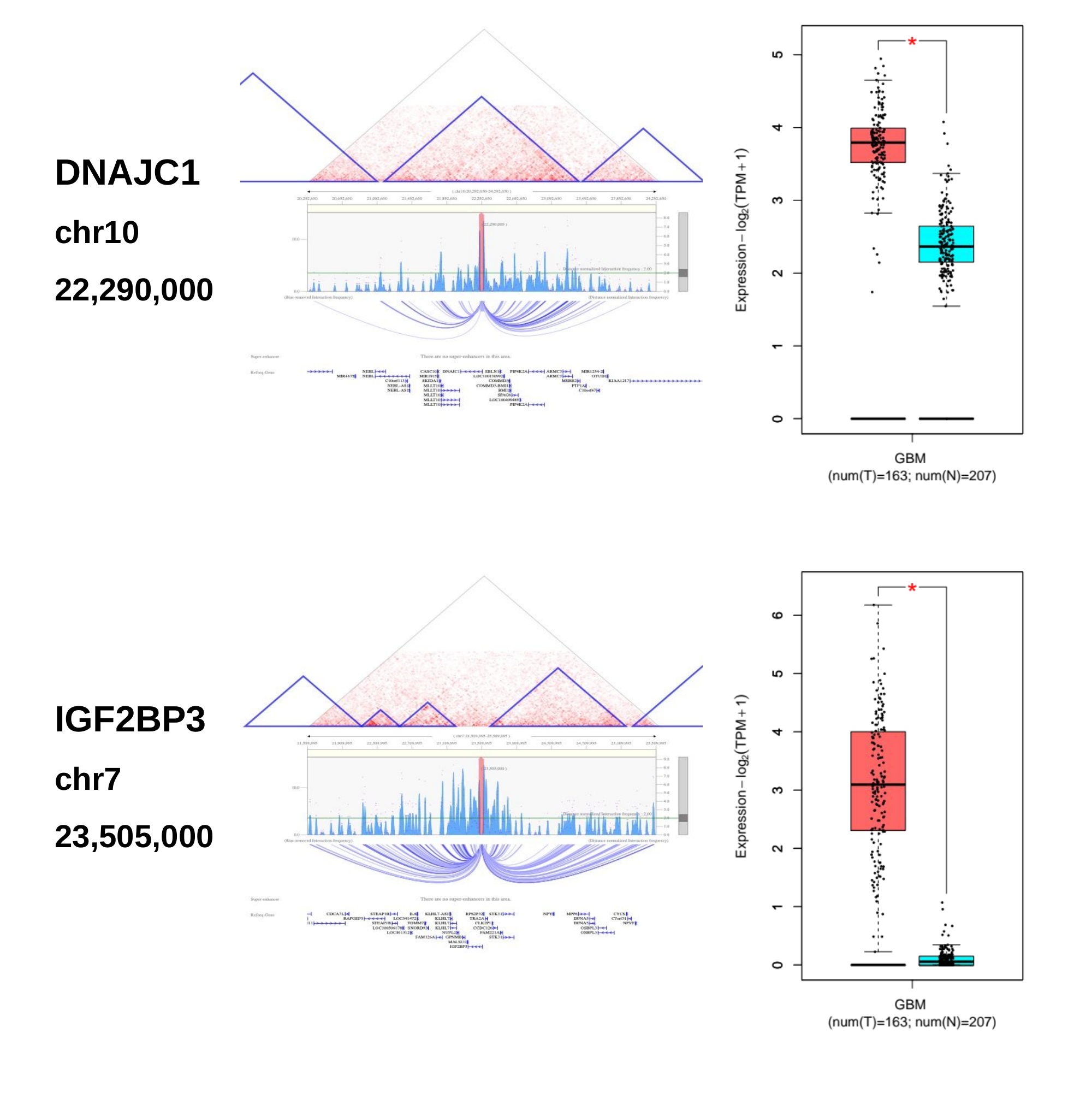

DNAJC1
chr10
22,290,000
IGF2BP3
chr7
23,505,000

## Slide 5
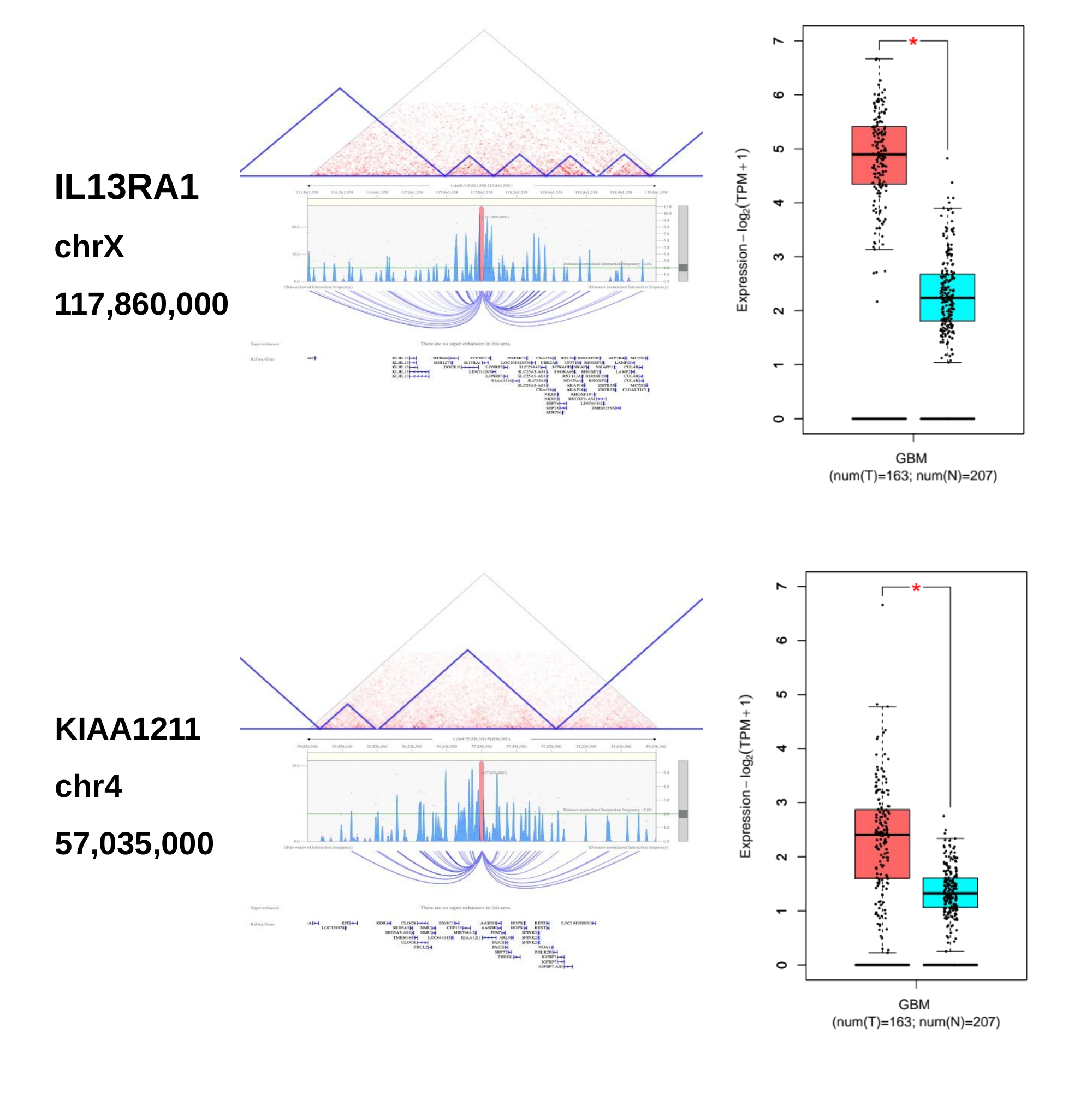

IL13RA1
chrX
117,860,000
KIAA1211
chr4
57,035,000

## Slide 6
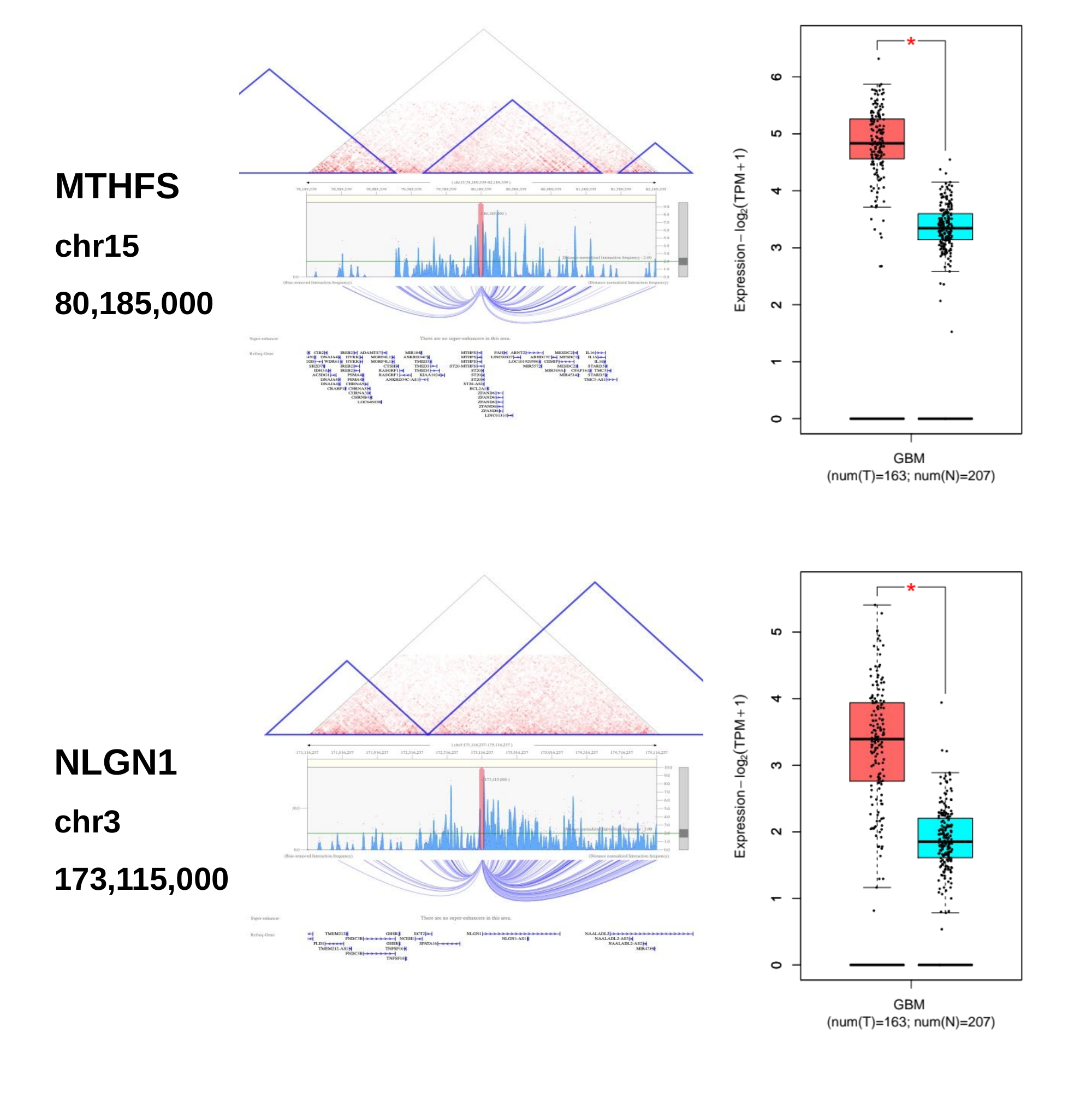

MTHFS
chr15
80,185,000
NLGN1
chr3
173,115,000

## Slide 7
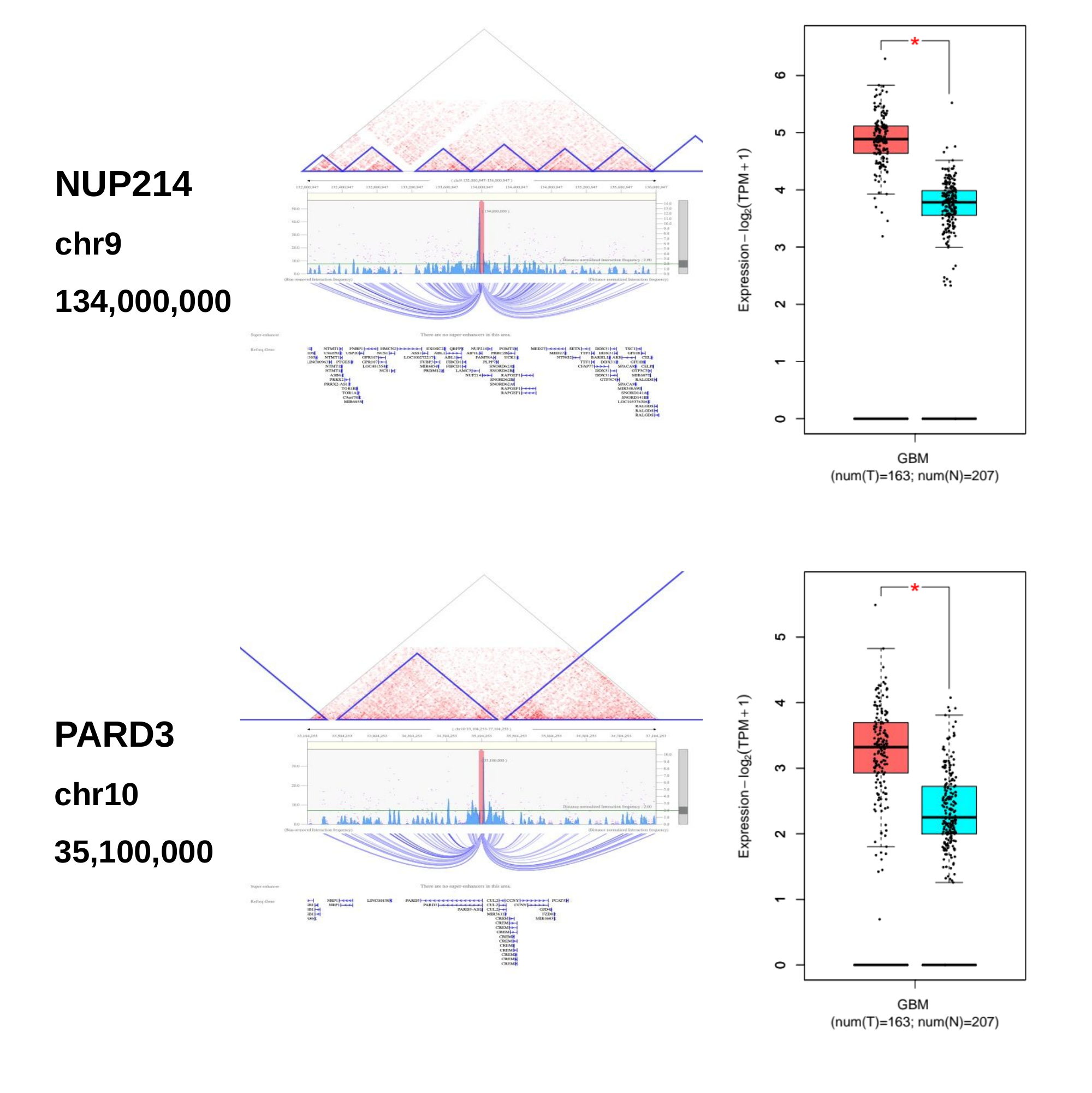

NUP214
chr9
134,000,000
PARD3
chr10
35,100,000

## Slide 8
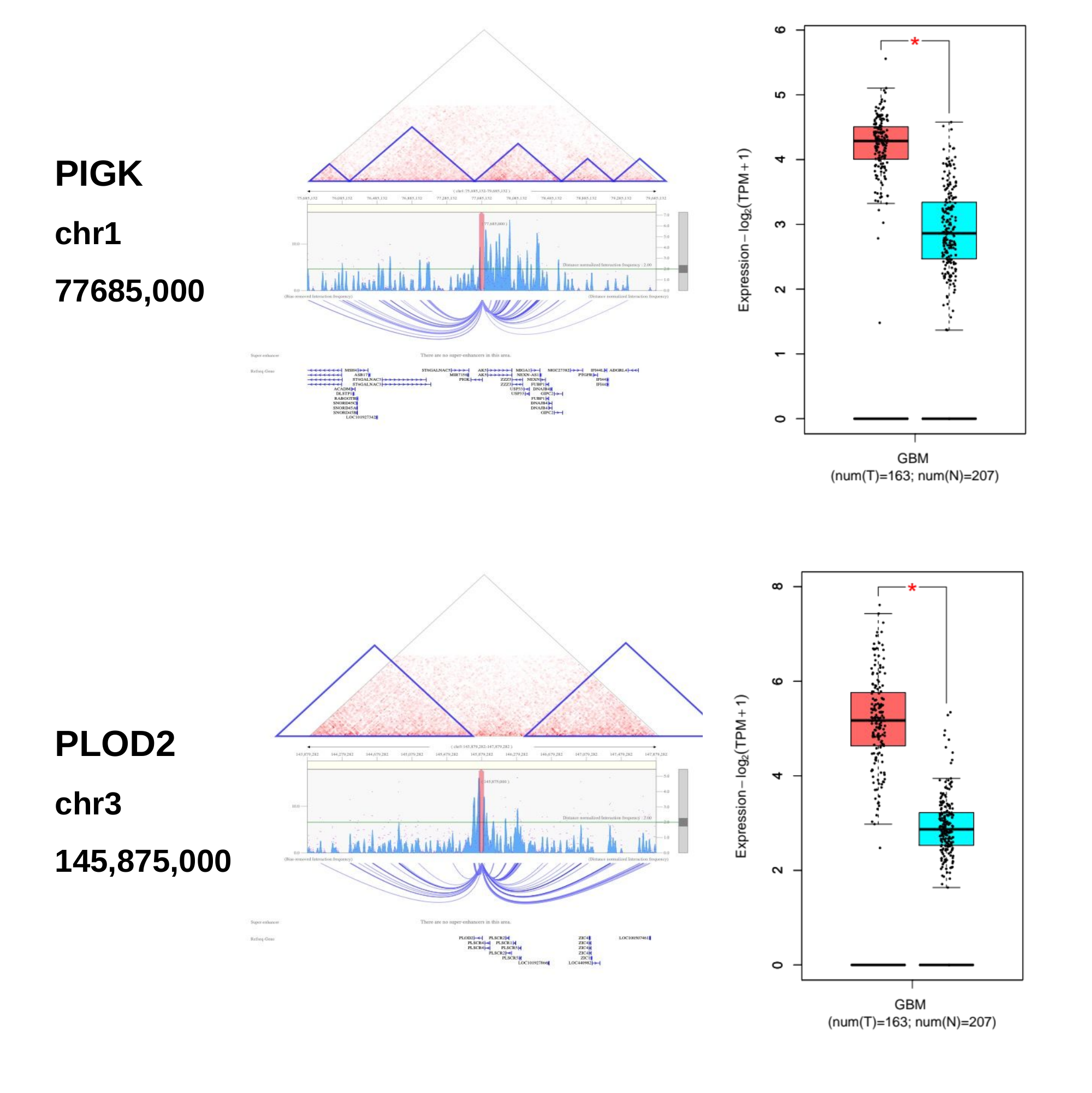

PIGK
chr1
77685,000
PLOD2
chr3
145,875,000

## Slide 9
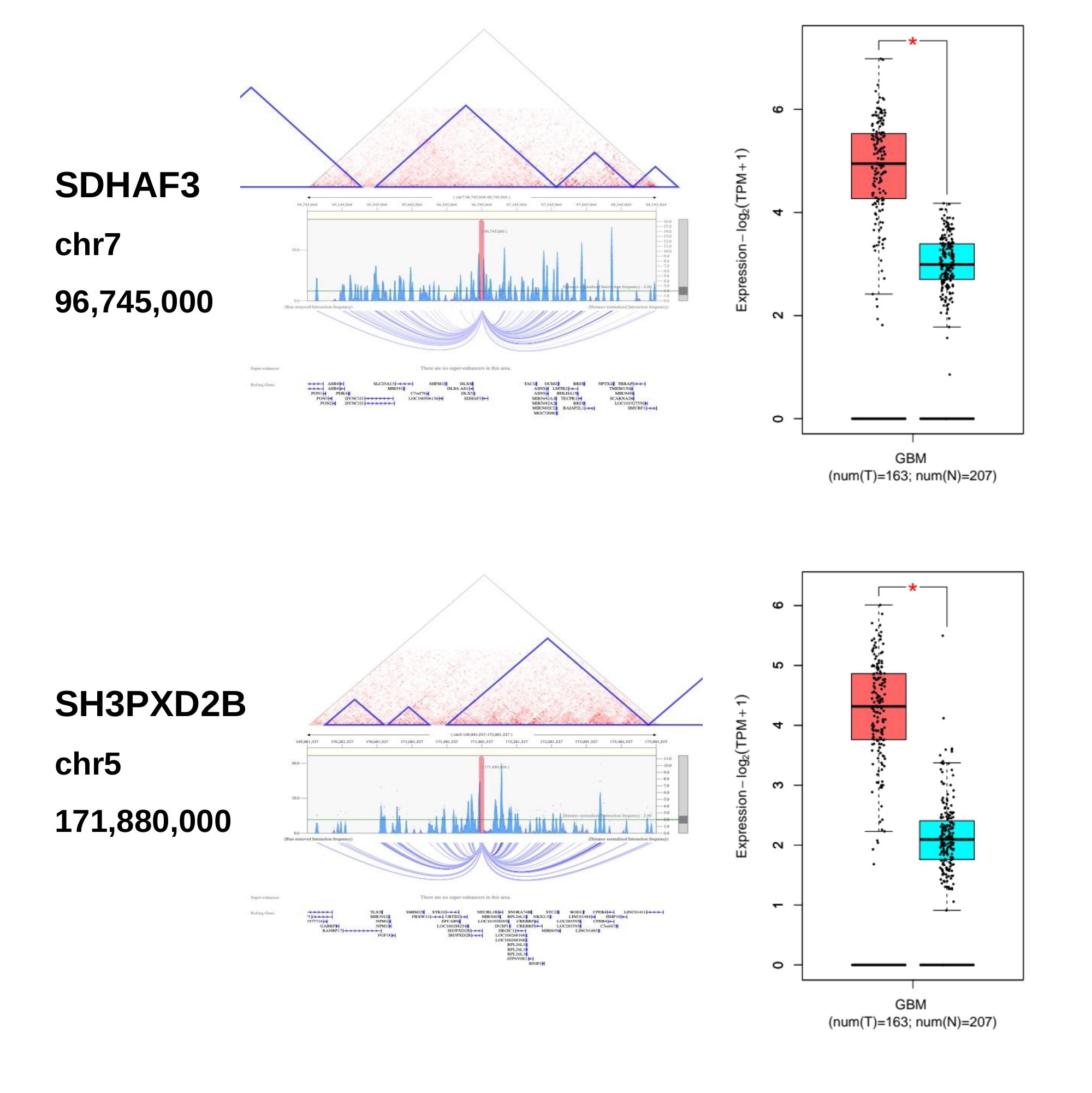

SDHAF3
chr7
96,745,000
SH3PXD2B
chr5
171,880,000

## Slide 10
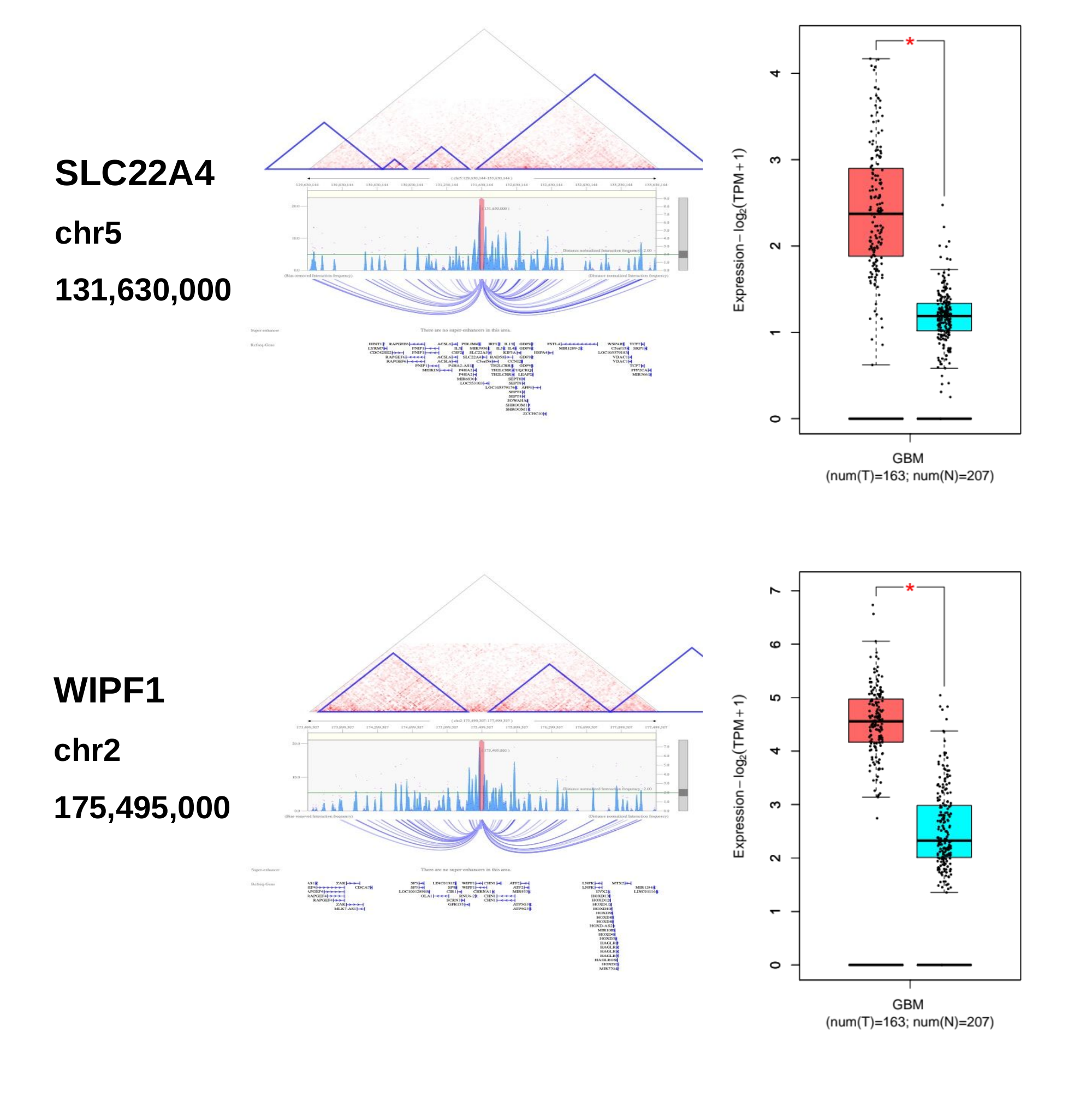

SLC22A4
chr5
131,630,000
WIPF1
chr2
175,495,000

## Slide 11
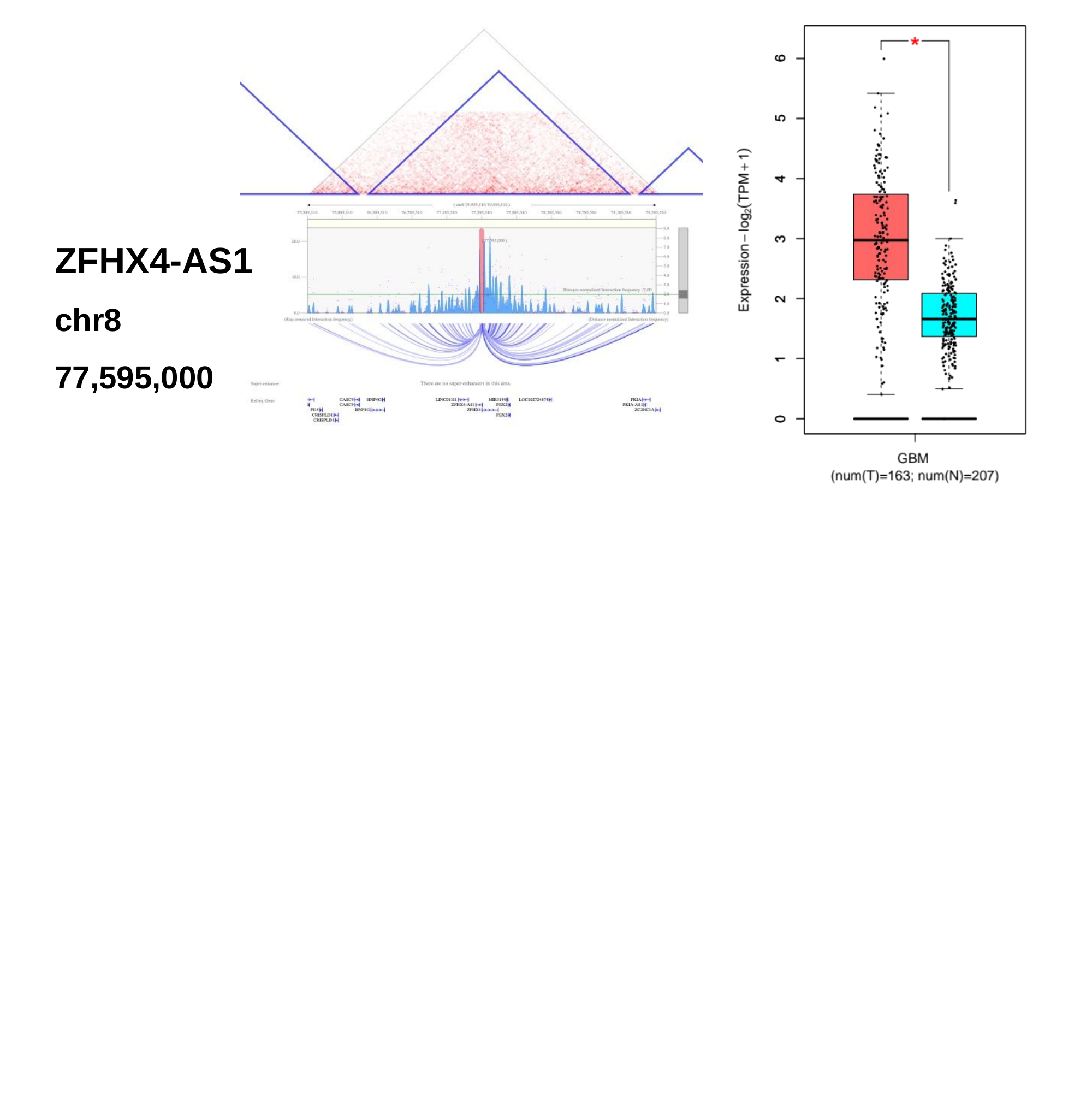

ZFHX4-AS1
chr8
77,595,000

## Slide 12
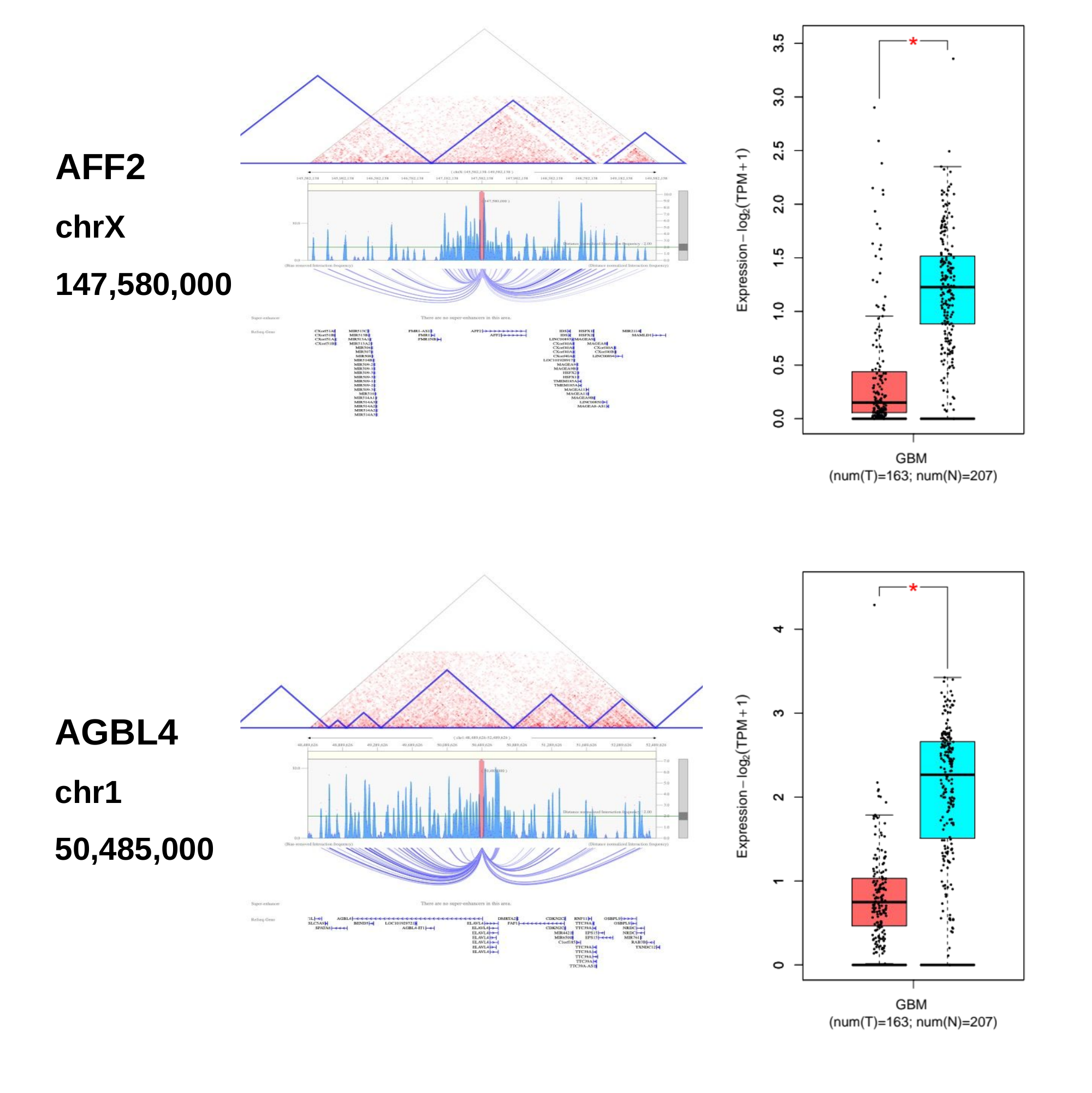

AFF2
chrX
147,580,000
AGBL4
chr1
50,485,000

## Slide 13
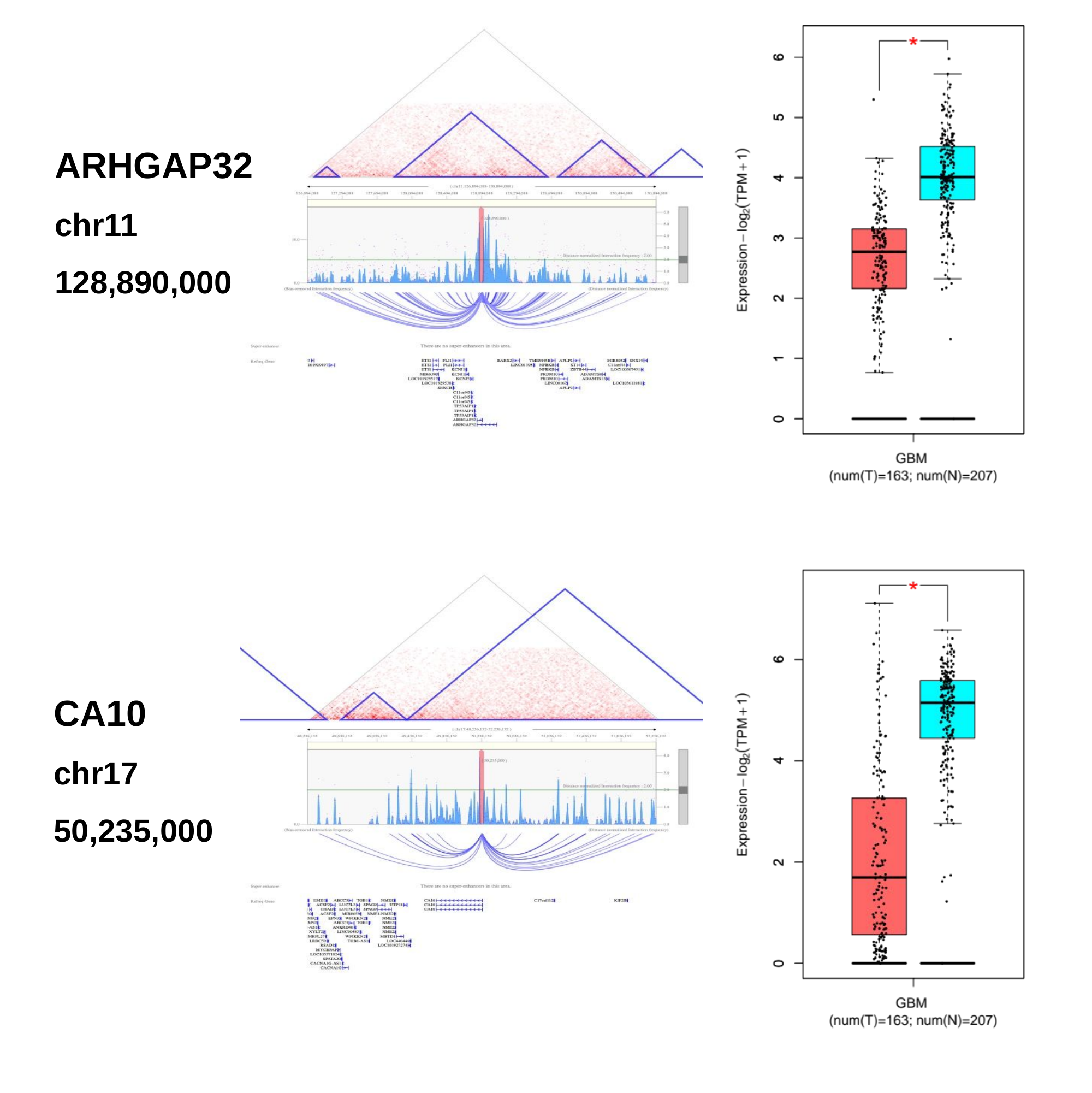

ARHGAP32
chr11
128,890,000
CA10
chr17
50,235,000

## Slide 14
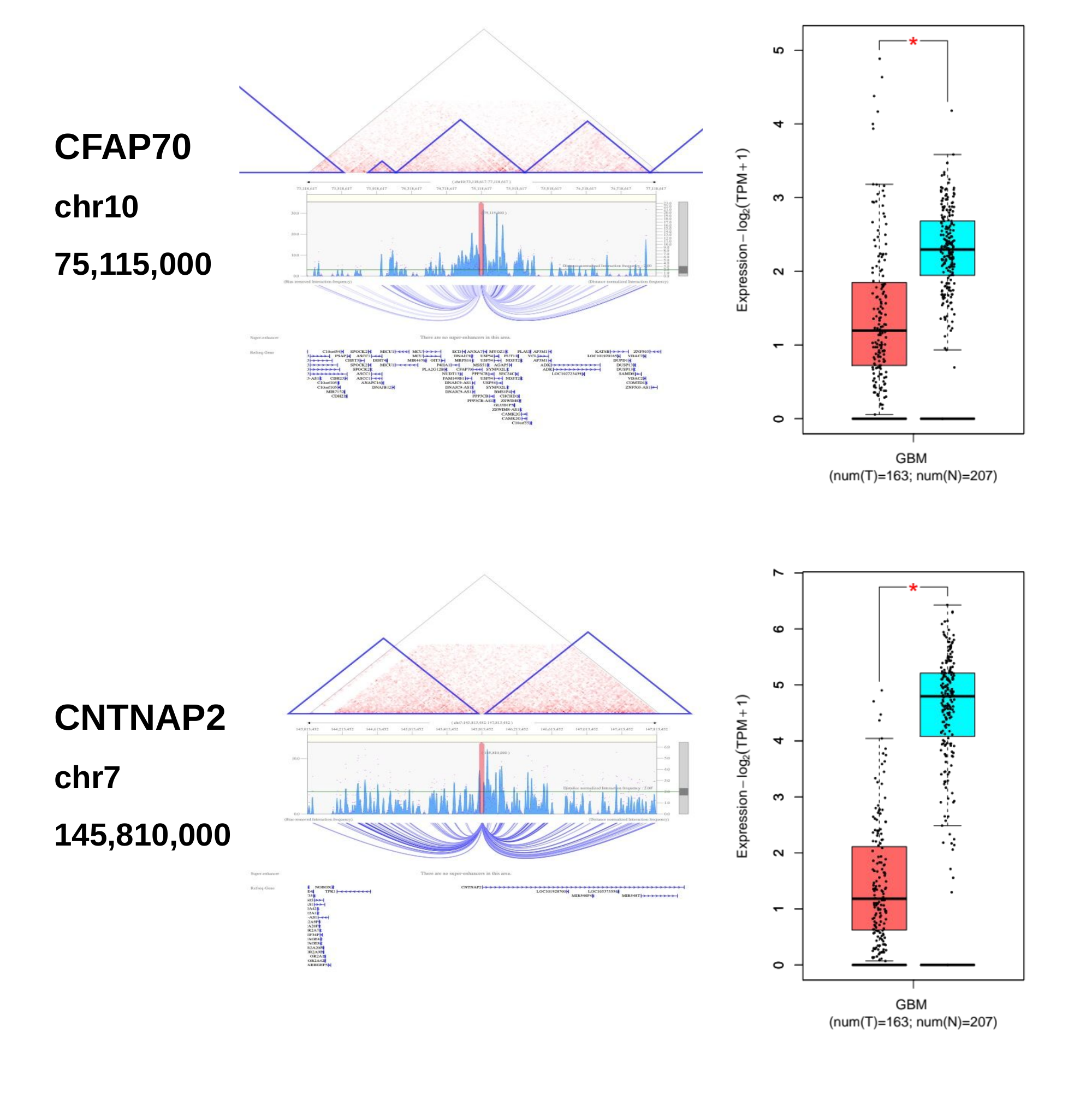

CFAP70
chr10
75,115,000
CNTNAP2
chr7
145,810,000

## Slide 15
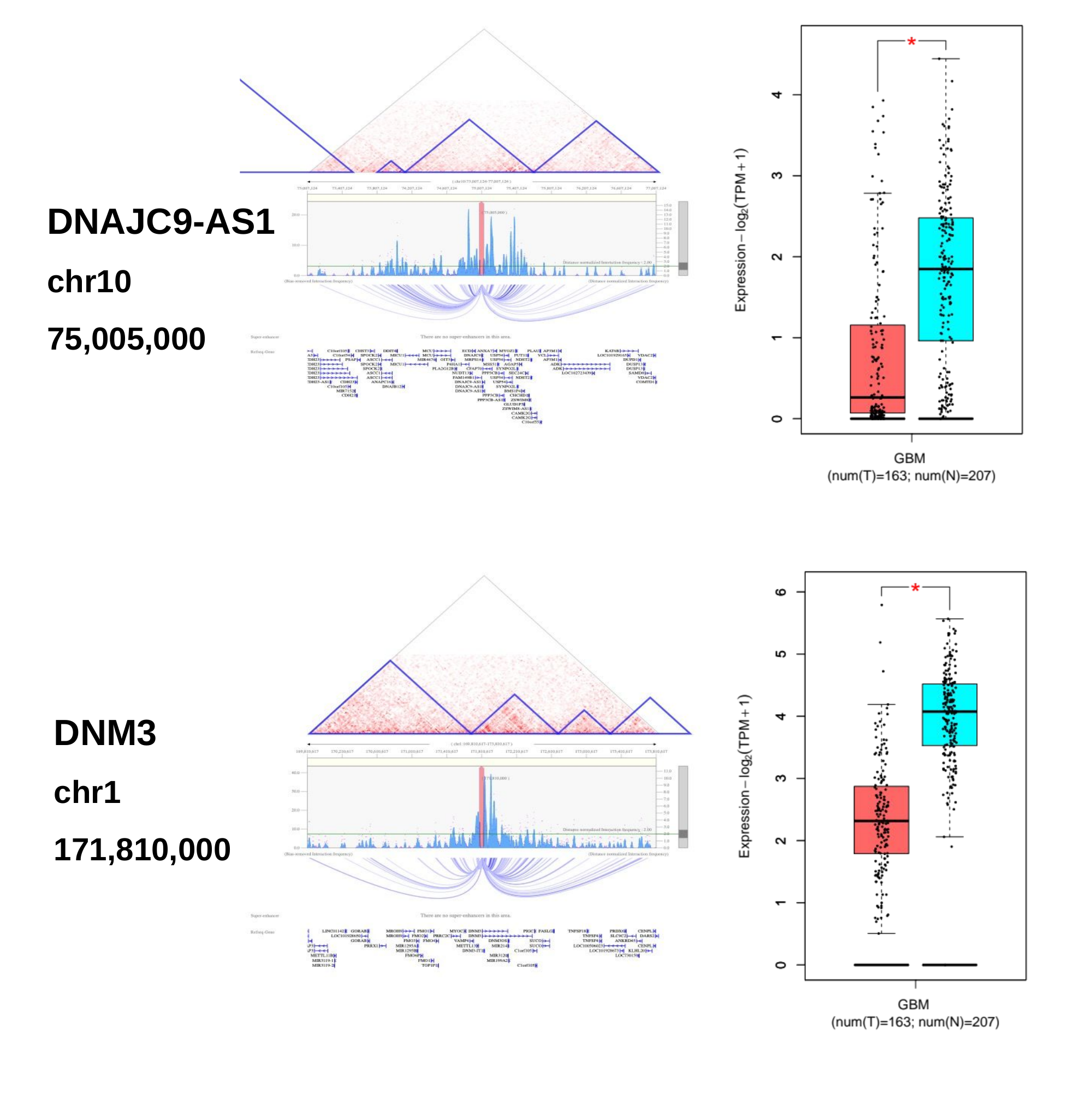

DNAJC9-AS1
chr10
75,005,000
DNM3
chr1
171,810,000

## Slide 16
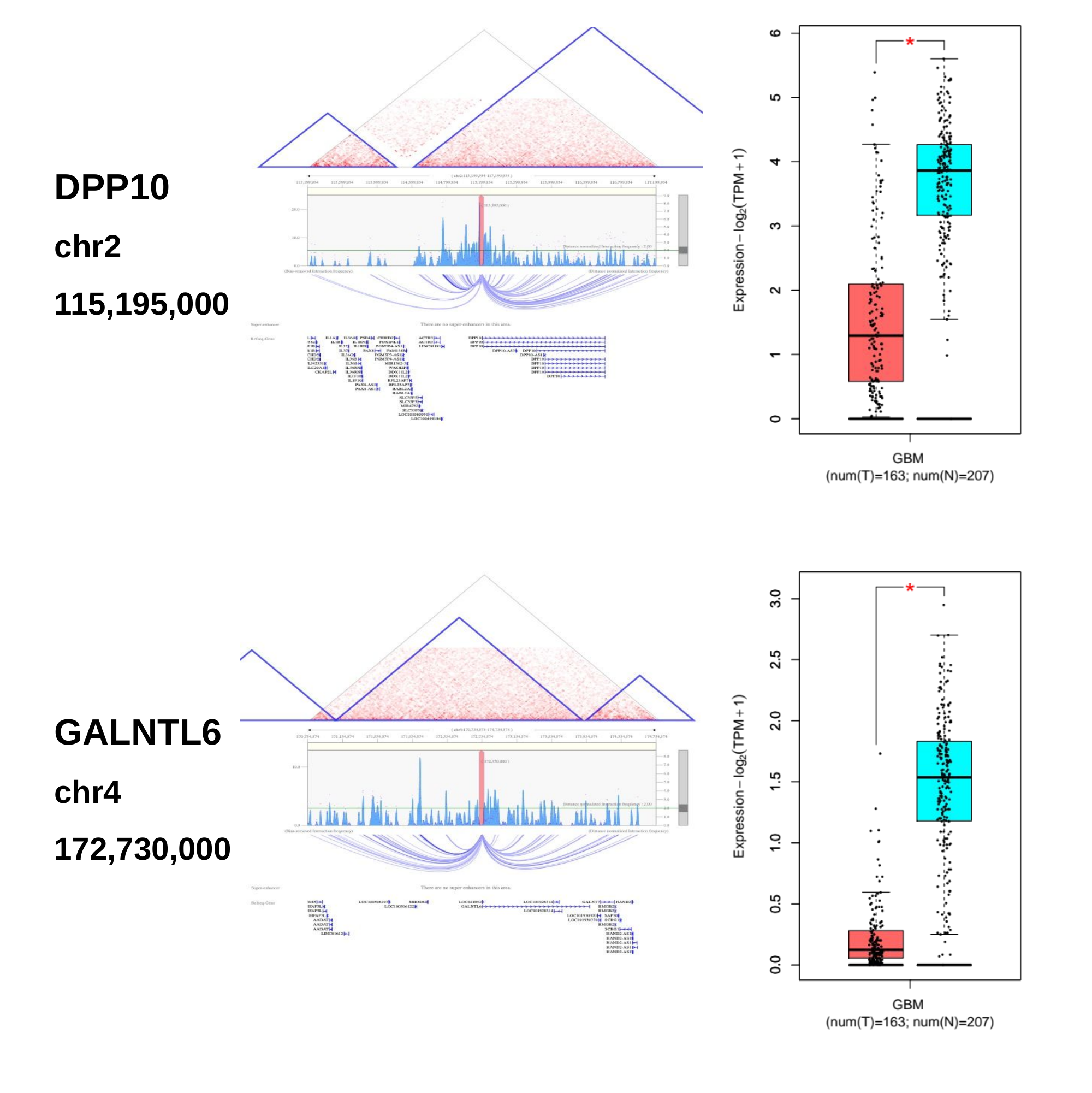

DPP10
chr2
115,195,000
GALNTL6
chr4
172,730,000

## Slide 17
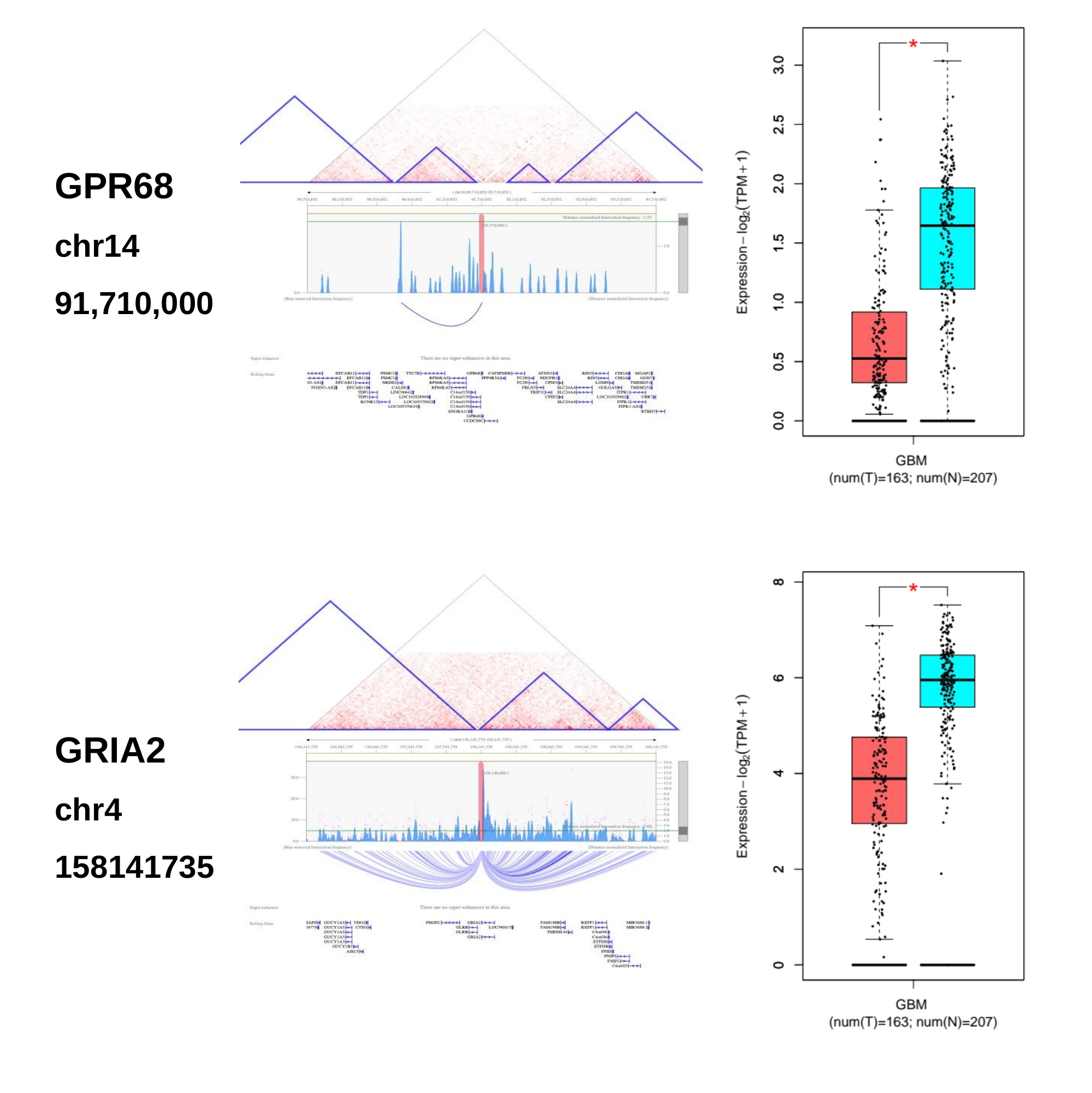

GPR68
chr14
91,710,000
GRIA2
chr4
158141735

## Slide 18
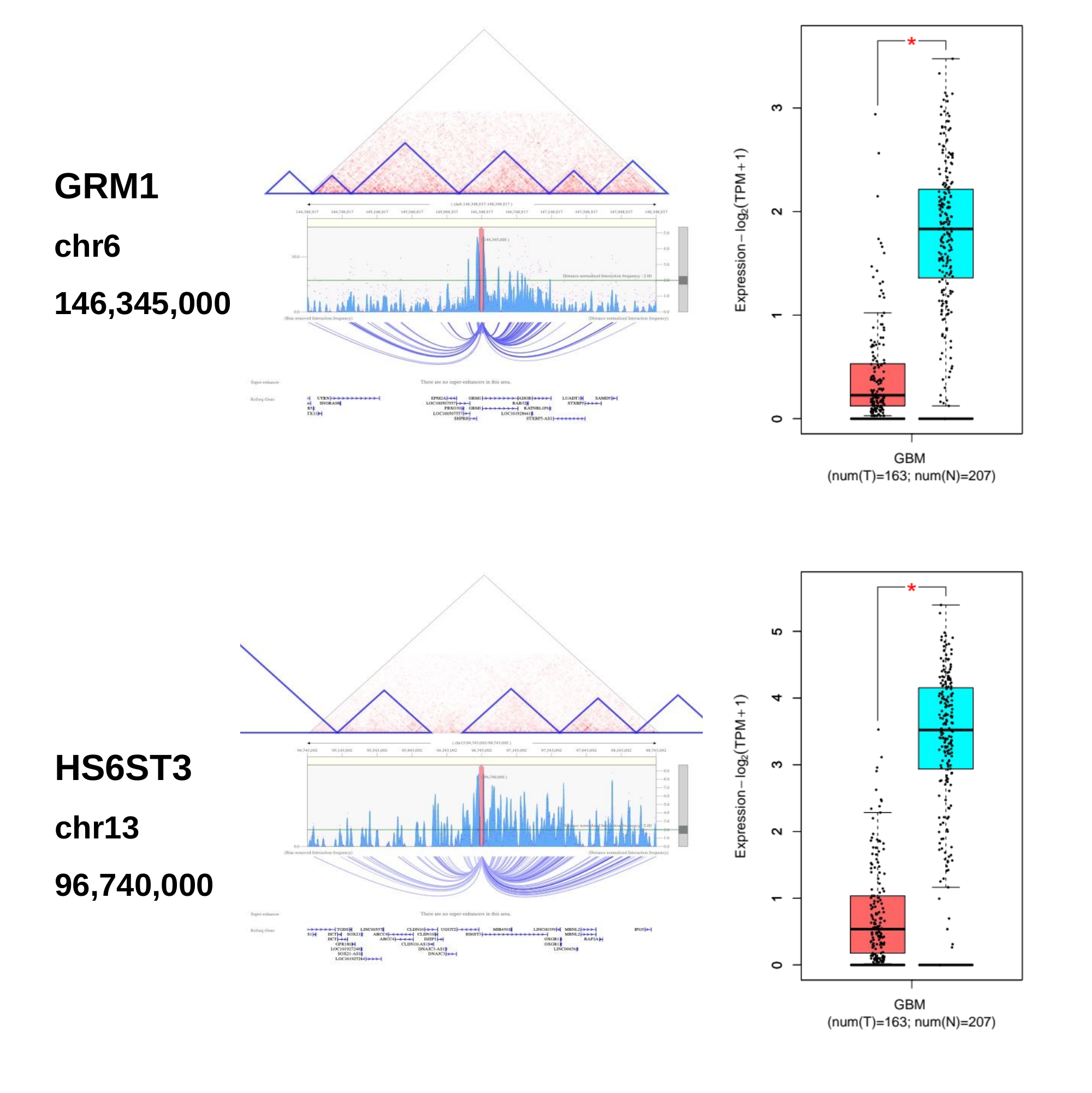

GRM1
chr6
146,345,000
HS6ST3
chr13
96,740,000

## Slide 19
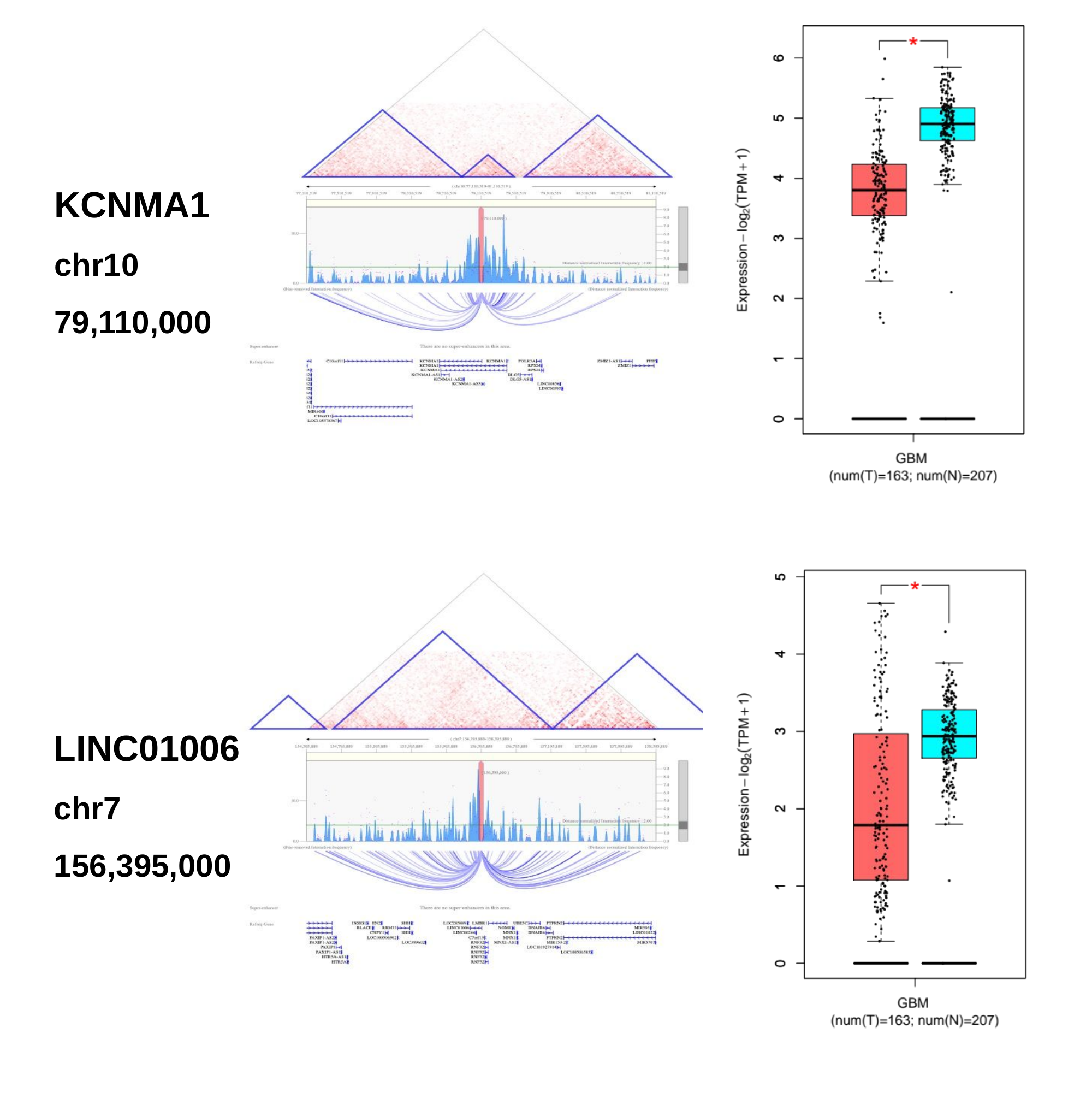

KCNMA1
chr10
79,110,000
LINC01006
chr7
156,395,000

## Slide 20
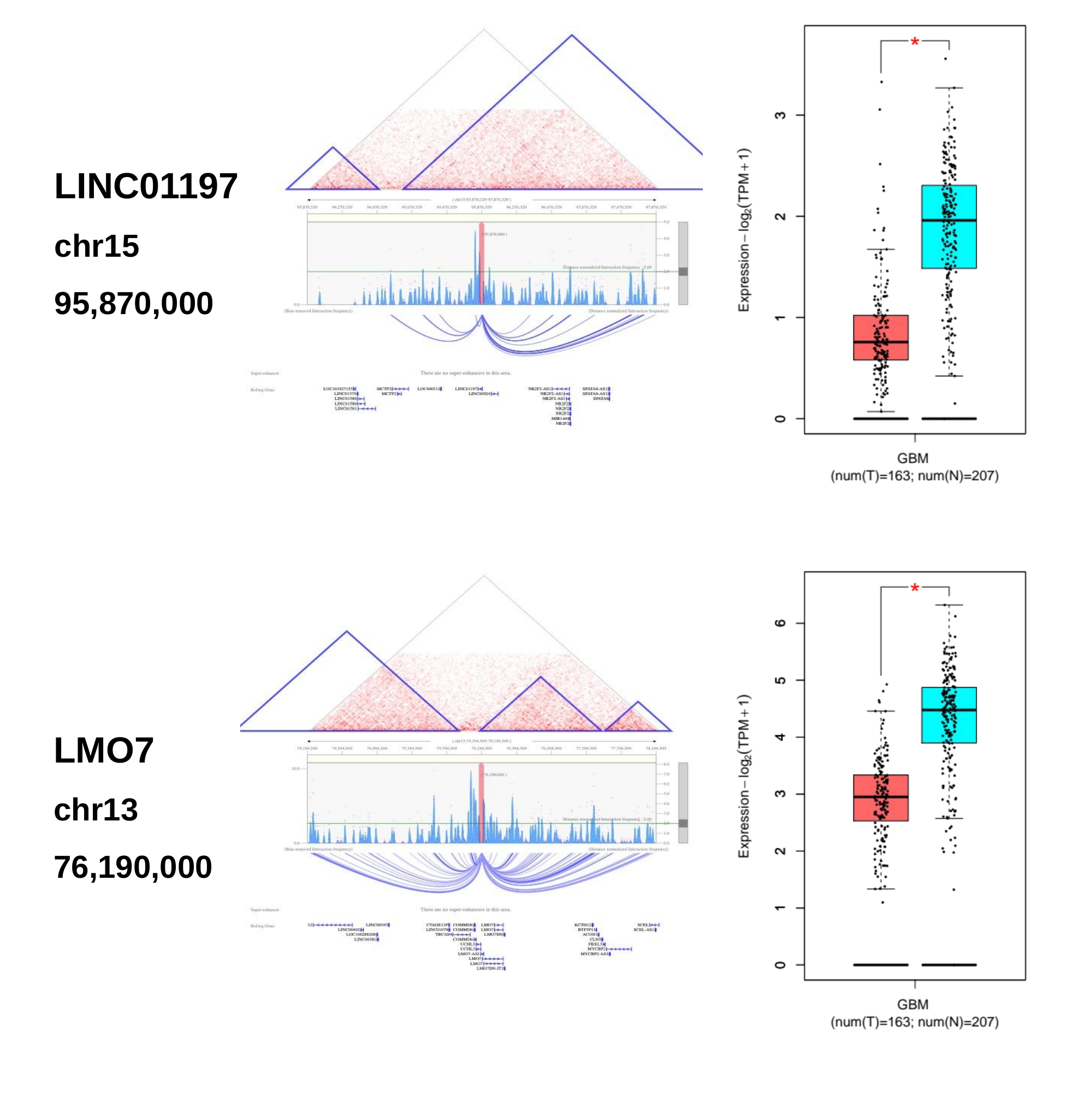

LINC01197
chr15
95,870,000
LMO7
chr13
76,190,000

## Slide 21
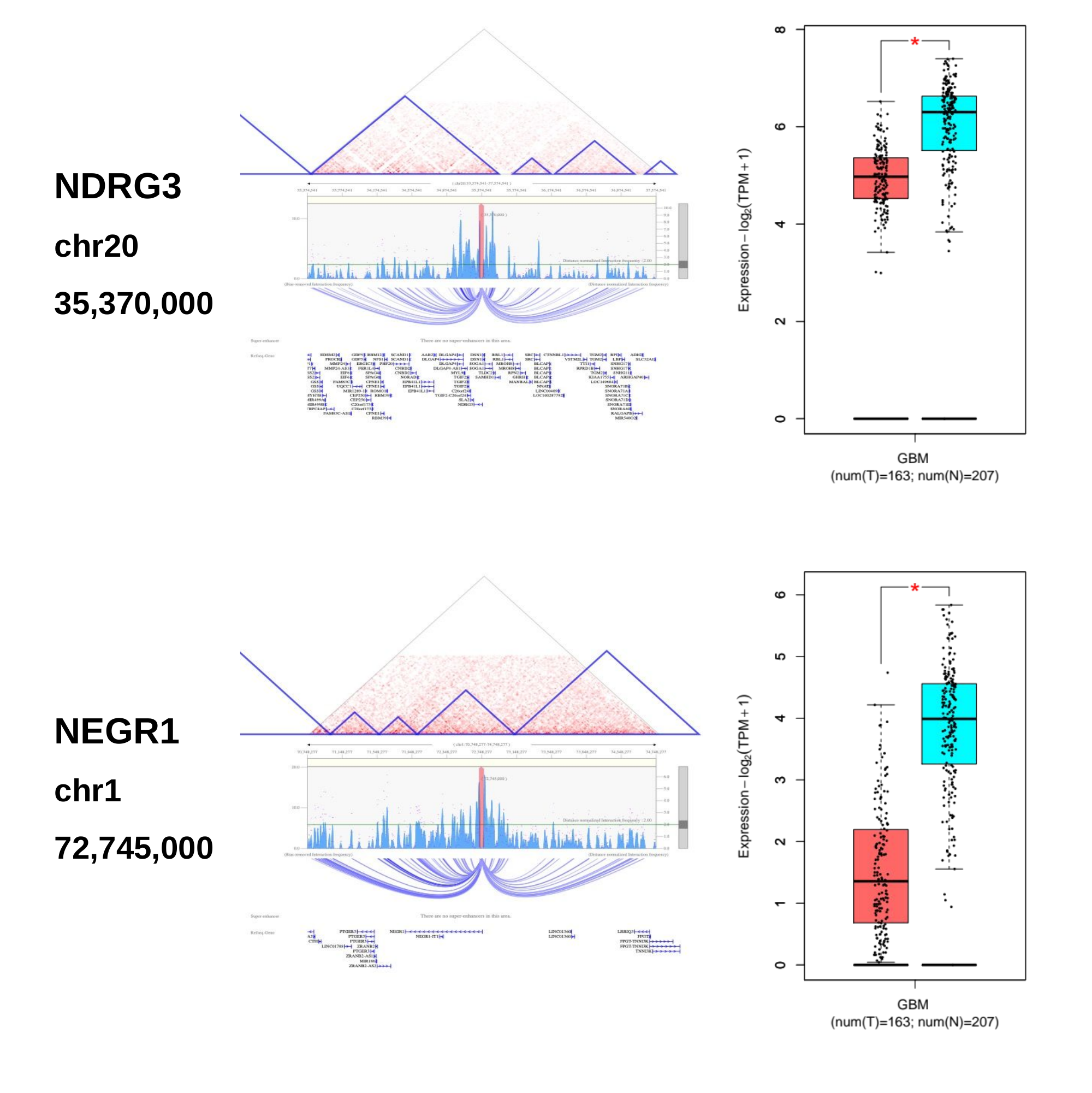

NDRG3
chr20
35,370,000
NEGR1
chr1
72,745,000

## Slide 22
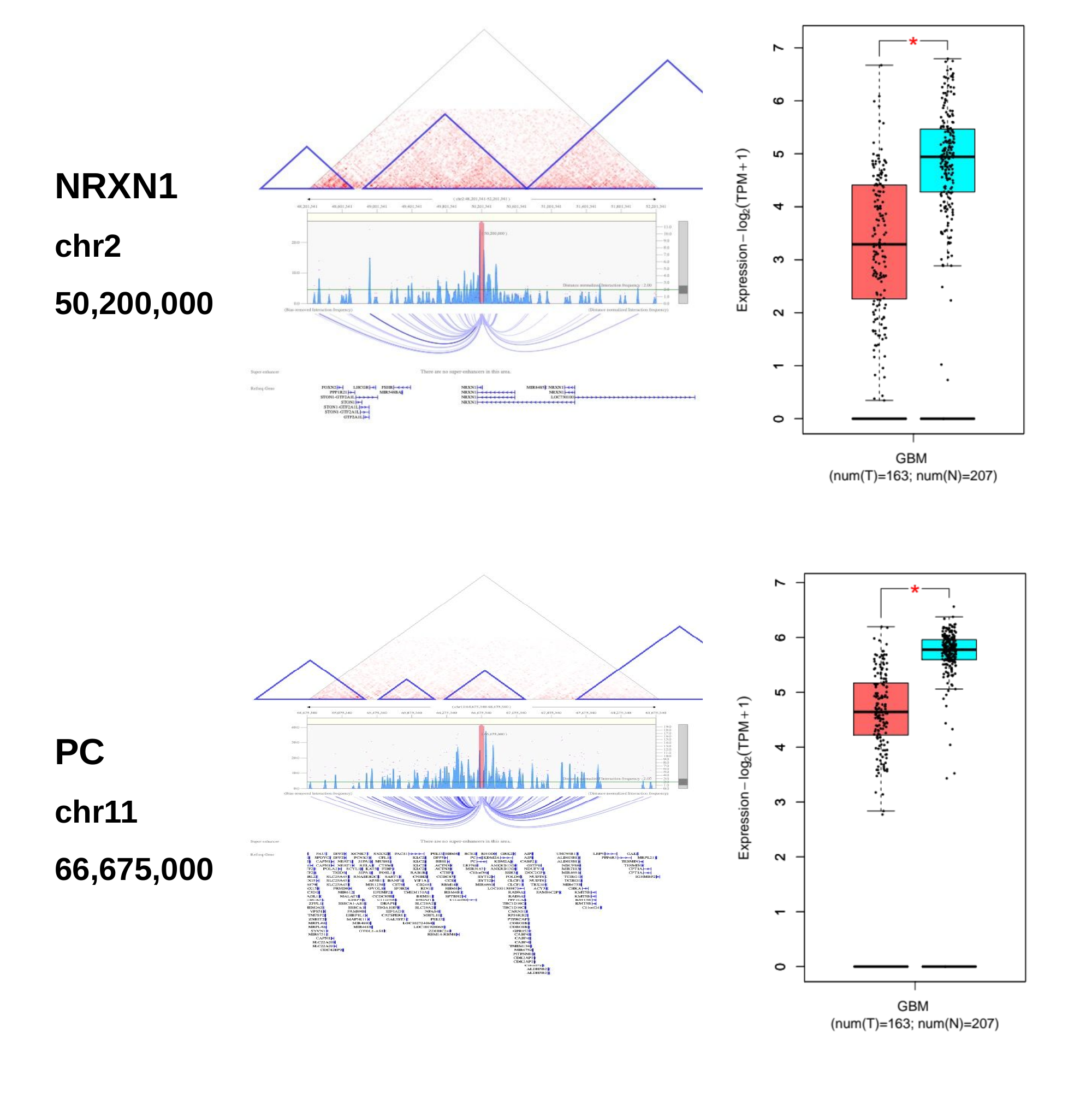

NRXN1
chr2
50,200,000
PC
chr11
66,675,000

## Slide 23
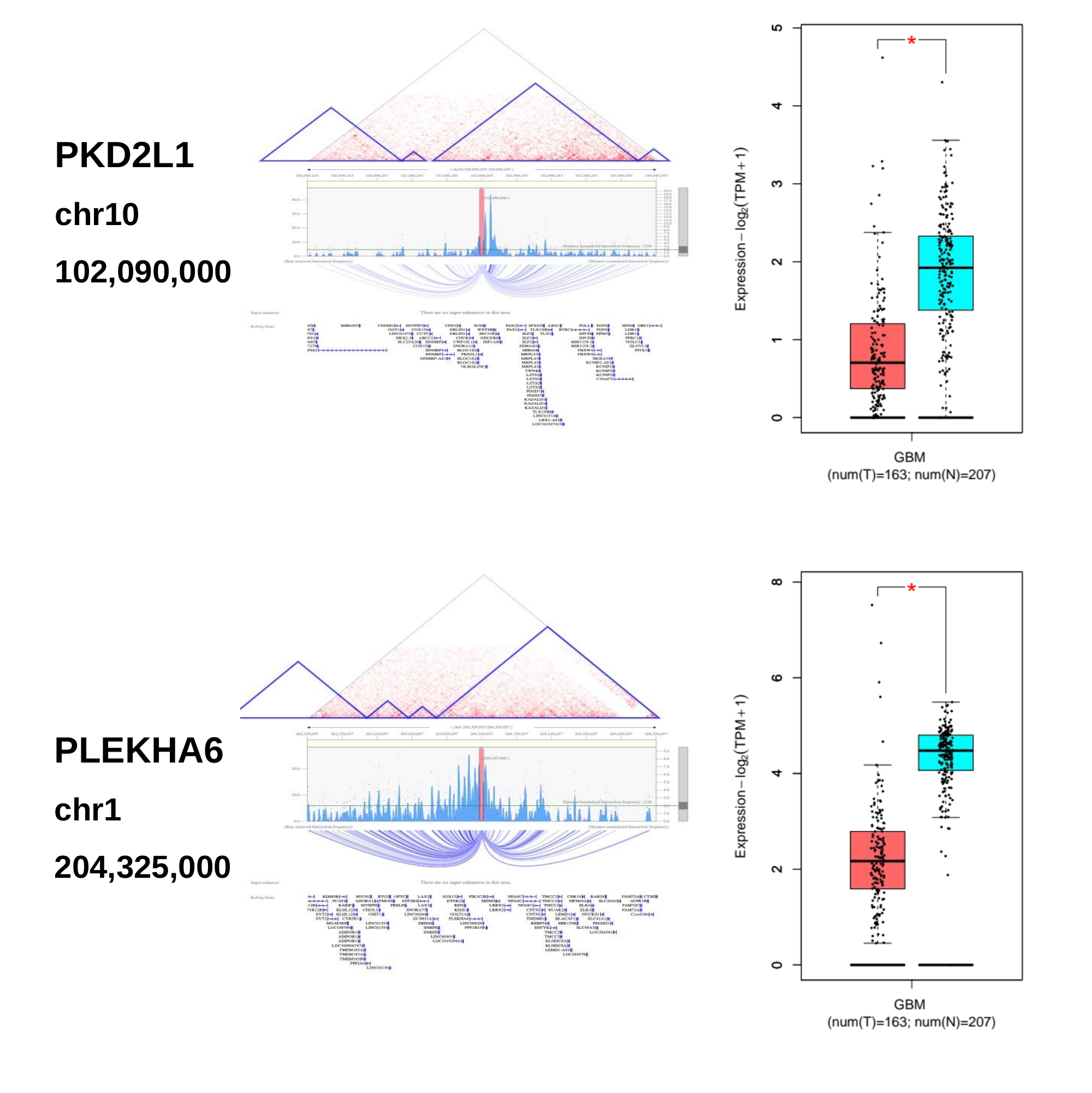

PKD2L1
chr10
102,090,000
PLEKHA6
chr1
204,325,000

## Slide 24
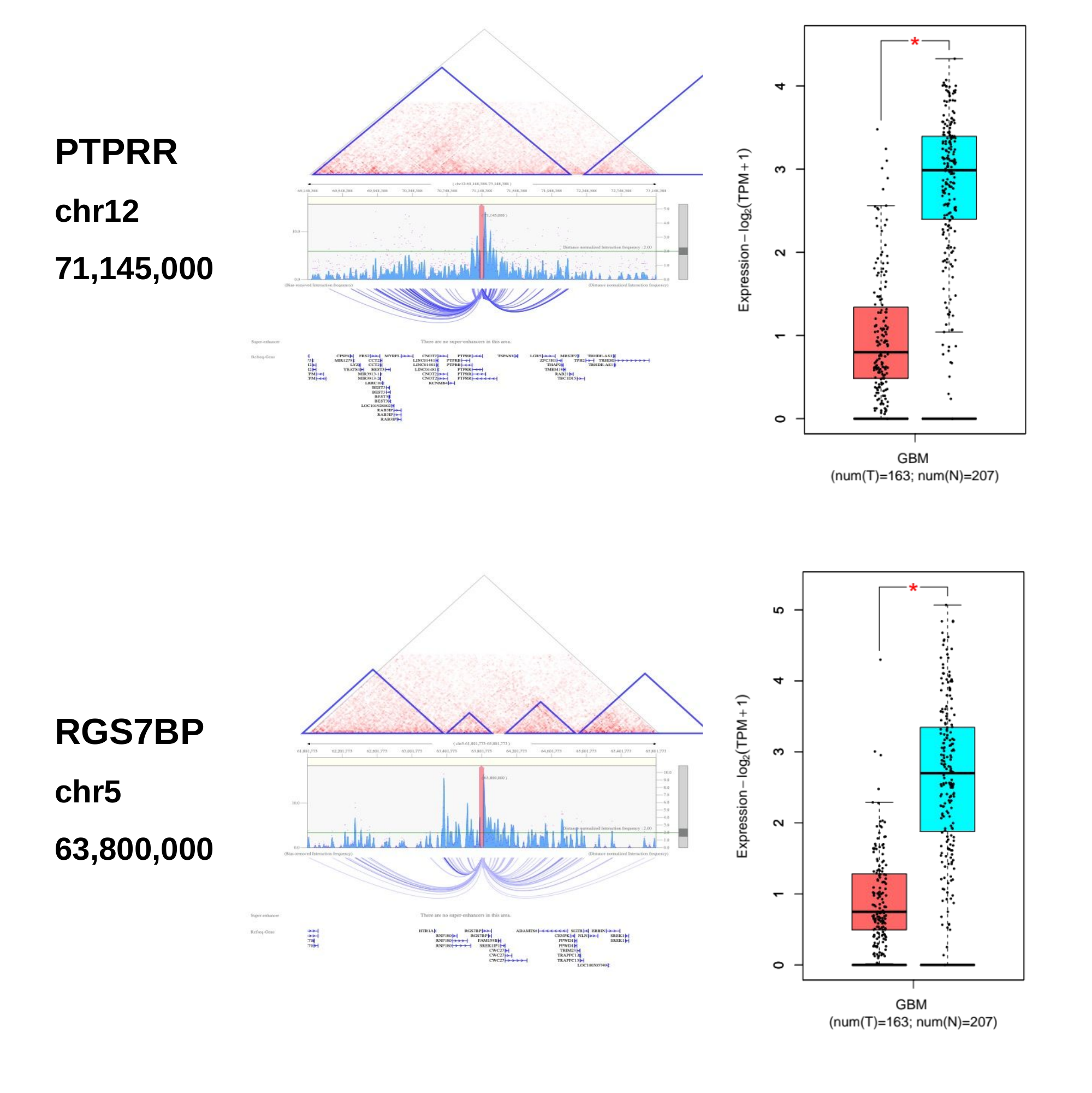

PTPRR
chr12
71,145,000
RGS7BP
chr5
63,800,000

## Slide 25
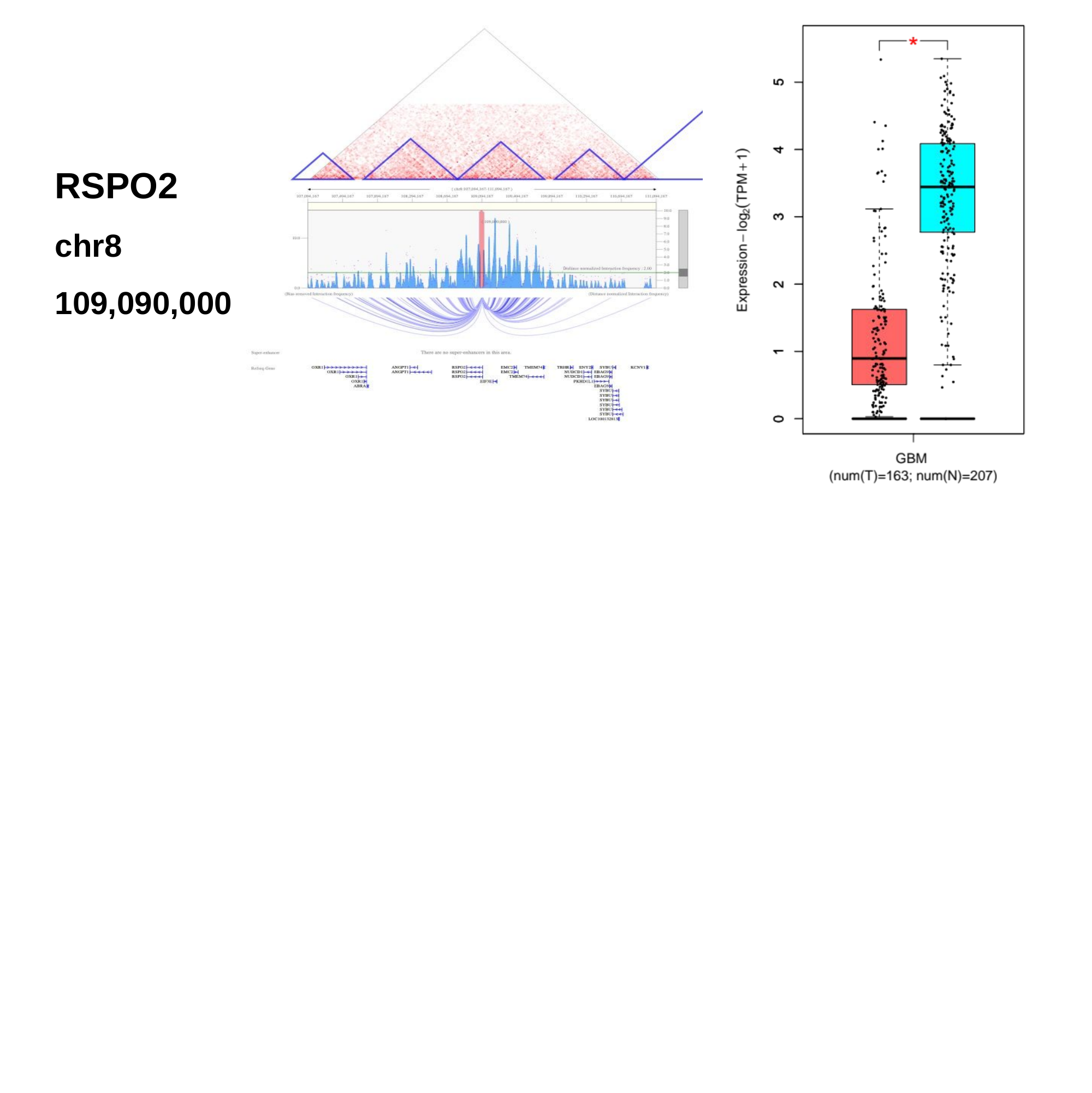

RSPO2
chr8
109,090,000
